# Lithographically Controlled Liquid Metal Diffusion in Graphene: Fabrication and Magnetotransport Signatures of Superconductivity

**DOI:** 10.1002/adma.202511992

**Published:** 2025-10-30

**Authors:** Stefan Wundrack, Marc Bothe, Marcelo Jaime, Kathrin Küster, Markus Gruschwitz, Yefei Yin, Zamin Mamiyev, Philip Schädlich, Bharti Matta, Sawani Datta, Marius Eckert, Christoph Tegenkamp, Ulrich Starke, Rainer Stosch, Hans Werner Schumacher, Thomas Seyller, Klaus Pierz, Teresa Tschirner, Andrey Bakin

**Affiliations:** ^1^ Physikalisch‐Technische Bundesanstalt Bundesallee 100 38116 Braunschweig Germany; ^2^ Institut für Halbleitertechnik Technische Universität Braunschweig Hans‐Sommer Straße 66 D‐38106 Braunschweig Germany; ^3^ Laboratory of Emerging Nanometrology (LENA) der Technischen Universität Braunschweig Langer Kamp 6 a/b 38106 Braunschweig Germany; ^4^ Institut für Physik Technische Universität Chemnitz Reichenhainer Str. 70 09126 Chemnitz Germany; ^5^ Max‐Planck‐Institut für Festkörperforschung Heisenbergstraße 1 70569 Stuttgart Germany

**Keywords:** confinement, graphene, Hall bar fabrication, intercalation, proximity, superconductivity

## Abstract

Metal intercalation in epitaxial graphene enables the emergence of proximity‐induced superconductivity and modified quantum transport properties. However, systematic transport studies of intercalated graphene have been hindered by challenges in device fabrication, including processing‐induced deintercalation and instability under standard lithographic techniques. Here, a lithographically controlled intercalation approach is introduced that enables the scalable fabrication of gallium‐intercalated quasi‐freestanding bilayer graphene (QFBLG) Hall bar devices. By integrating lithographic structuring with subsequent intercalation through dedicated intercalation channels, this method ensures precise control over metal incorporation while preserving device integrity. Magnetotransport measurements reveal superconductivity with a critical temperature $T_{c}^{\mathrm{onset}}$≈ 3.5 K and the occurrence of a transverse resistance, including both symmetric and antisymmetric field components, which is attributed to the symmetric‐in‐field component of non‐uniform currents. These results establish an advanced fabrication method for intercalated graphene devices, providing access to systematic investigations of confined 2D superconductivity and emergent electronic phases in van der Waals heterostructures.

## Graphical abstract

Lithography‐guided intercalation creates a proximitized 2D superconducting Ga layer under graphene Hall bars via liquid‐metal intercalation. The image shows a Hall bar where Ga atoms from the liquid phase enter through patterned intercalation channels beneath quasi‐freestanding bilayer graphene, forming a confined 2D metal that demonstrates superconductivity near 3.5 K and suppresses the quantum Hall effect in the intercalated graphene.

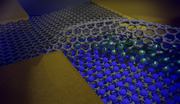

## 1 Introduction

The study of proximity effects in condensed matter physics has provided significant insights into how electronic properties can be tuned in materials. The proximity effect, particularly in superconducting systems, plays a crucial role in enabling and controlling quantum phenomena in two‐dimensional (2D) materials. Over the past few decades, considerable efforts have been made to induce superconductivity in non‐superconducting systems, including graphene, through various proximity effects.^[^ ^1^, ^2^, ^3^ ^]^

Graphene, a single layer of carbon atoms arranged in a honeycomb lattice,^[^ ^4^ ^]^ has emerged as a model system for investigating quantum transport phenomena. While pristine graphene lacks an intrinsic superconducting gap due to its Dirac‐like band structure, several approaches have been developed to induce superconductivity, including intercalation with metallic and superconducting elements,^[^ ^5^ ^]^ and the use of heterostructures with topological insulators.^[^ ^6^ ^]^ Among these, metal intercalated epitaxial graphene has gained significant interest due to its tunable electronic properties.^[^ ^7^, ^8^, ^9^, ^10^, ^11^ ^]^Here, a microscopic understanding of the graphene–metal intercalant coupling is essential for inducing superconductivity.

Einenkel and Efetov^[^ ^12^ ^]^ examined theoretically the role of charge carrier concentration and of electron‐phonon interactions in graphene to facilitate intrinsic superconductivity. They concluded that superconductivity in heavily electron‐doped graphene becomes favorable when approaching the van Hove singularity (VHS) with electron densities up to 10^14^ cm^−2^. While experimental verification remains pending, their theoretical predictions align with recent observations of enhanced superconducting coupling in gold‐intercalated graphene.^[^ ^5^ ^]^ Furthermore, the realization of *d*‐wave superconductivity has been investigated by employing different metal dopants to graphene (e.g., terbium (Tb), lithium (Li) and cesium (Cs)).^[^ ^13^ ^]^ The theoretical finding reveals that alkali dopants such as Li and Cs alkali intercalants impose ($\sqrt{3}$ × $\sqrt{3}$) or (2 × 2) superlattices, affecting graphenes lattice symmetry and thus resulting into a disturbance of *d*‐wave states leading to instability of superconductivity. In contrast, Tb intercalation injects sufficient carriers to place the Fermi level at the extended VHS, while essentially preserving the lattice symmetry.^[^ ^13^ ^]^ Within a random‐phase‐approximation treatment this configuration supports a robust chiral *d*+*id* superconducting state with predicted *T*_c_​ up to ≈0.6 K.^[^ ^13^ ^]^ Thus, only symmetry‐preserving rare‐earth intercalants like Tb or potentially Gd and Yb emerge as promising routes toward realizing *d*‐wave superconductivity in single‐layer graphene.^[^ ^13^ ^]^

In addition to intercalation, twisted‐bilayer graphene (TBG) has emerged as a highly promising platform for investigating superconductivity. When two graphene layers are twisted at a magic angle (≈1.1°), flat electronic bands form, leading to strong electronic correlations that give rise to superconductivity.^[^ ^3^ ^]^ The discovery of superconductivity in TBG has opened a new avenue for understanding unconventional superconductors and offers possibilities for engineering tunable superconducting states in graphene‐based systems.

Moreover, recent work on gallium‐intercalated graphene heterostructures has demonstrated their potential in proximity‐induced superconductivity.^[^ ^6^, ^14^ ^]^

A significant limitation in the study of intercalated graphene systems arises in the context of magnetotransport measurements. While techniques such as angle‐resolved photoemission spectroscopy (ARPES) and scanning tunneling microscopy (STM) have provided crucial insights into the electronic band structure and the density of states (DOS), these methods do not directly probe charge transport properties. This has hindered efforts to establish a clear correlation between intercalation‐induced band structure modifications and quantum transport phenomena, such as resistance characteristics, the suppression or modification of the quantum Hall effect (QHE), and the emergence of exotic quantum phases like the quantum spin Hall insulator (QSHI) or the anomalous quantum Hall effect (AQHE).

Beyond fundamental physics, precise transport characterization is essential for metrological applications of QHE, particularly as a quantum resistance standard.^[^ ^15^ ^]^ The QHE plays a central role in the traceability of fundamental units such as the ohm, farad, ampere, and kilogram in the revised International System of Units (SI).^[^ ^16^ ^]^ The ability to precisely control intercalation raises the question of whether new quantum effects, such as QSHI or AQHE, can emerge in metal‐intercalated graphene under high magnetic fields. Understanding these effects is crucial for advancing 2D quantum transport physics and assessing the potential of intercalated graphene for next‐generation quantum electrical standards.

To address this gap, our work presents a lithographical fabrication route of epitaxial graphene Hall bars, making intercalated graphene systems accessible for transport measurements. The proposed chip design preserves the integrity of the confined metal layers beneath graphene, enabling reliable magnetotransport experiments. Systematic magnetotransport measurements are carried out for investigations of superconductivity in gallium (Ga) intercalated quasi‐freestanding bilayer graphene on 4H‐SiC (SiC/2DGa/QFBLG), revealing a superconducting transition at $T_{c}^{\mathrm{onset}}$≈ 3.5 K, the absence of QHE above $T_{c}^{\mathrm{onset}},$ and the occurrence of a transverse resistance, which exhibits both symmetric and antisymmetric field components attributed to the symmetric‐in‐field component of non‐uniform currents. The ability to control the metal diffusion beneath graphene establishes a scalable platform for exploring proximity‐induced superconductivity and other transport phenomena in metal‐intercalated epitaxial graphene.

## 2 Results and Discussion

### 2.1 Design of Liquid Metal Intercalation of Epitaxial Graphene Hall Bars

**Figure 1:**
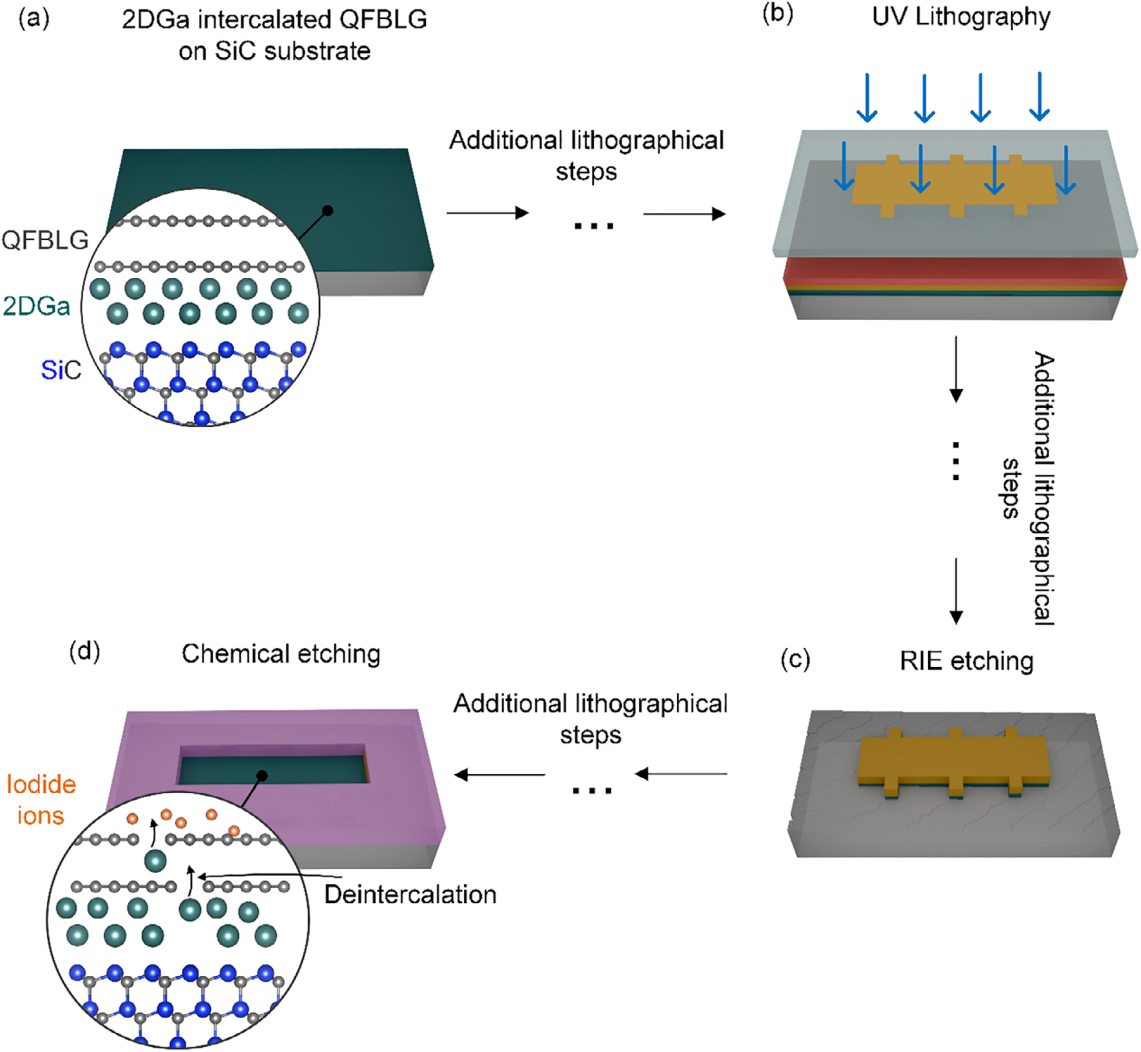
Schematic illustration of bottleneck in lithographical fabrication of Ga‐intercalated Hall bar devices. a) 2DGa‐intercalated quasi‐freestanding bilayer graphene (QFBLG) on a SiC substrate. b) UV lithography is performed to pattern the Hall bar geometry. c) Reactive ion etching (RIE) is applied to remove unwanted graphene, leaving the Hall bar intact. d) Chemical etching step leading to deintercalation of intercalated metals beneath QFBLG.

Usually, the fabrication of graphene devices is done via lithography after the metal intercalation process, as schematically shown in **Figure** **1**a–d. Due to the harsh conditions during reactive ion etching or chemical etching with potassium iodide (KI) or other etching chemicals, the intercalated metal layer is often removed (Figure 1d and Figure S2, Supporting Information). Chemical treatment with KI on Ga‐intercalated reference samples (Figure S4, Supporting Information) demonstrates deintercalation of Ga, evidencing the chemical instability of metal‐intercalated layers. Therefore, we introduce an alternative approach in which an entirely fabricated epitaxial graphene Hall bar device is intercalated with metal atoms in a subsequent step.^[^ ^17^ ^]^

**Figure 2:**
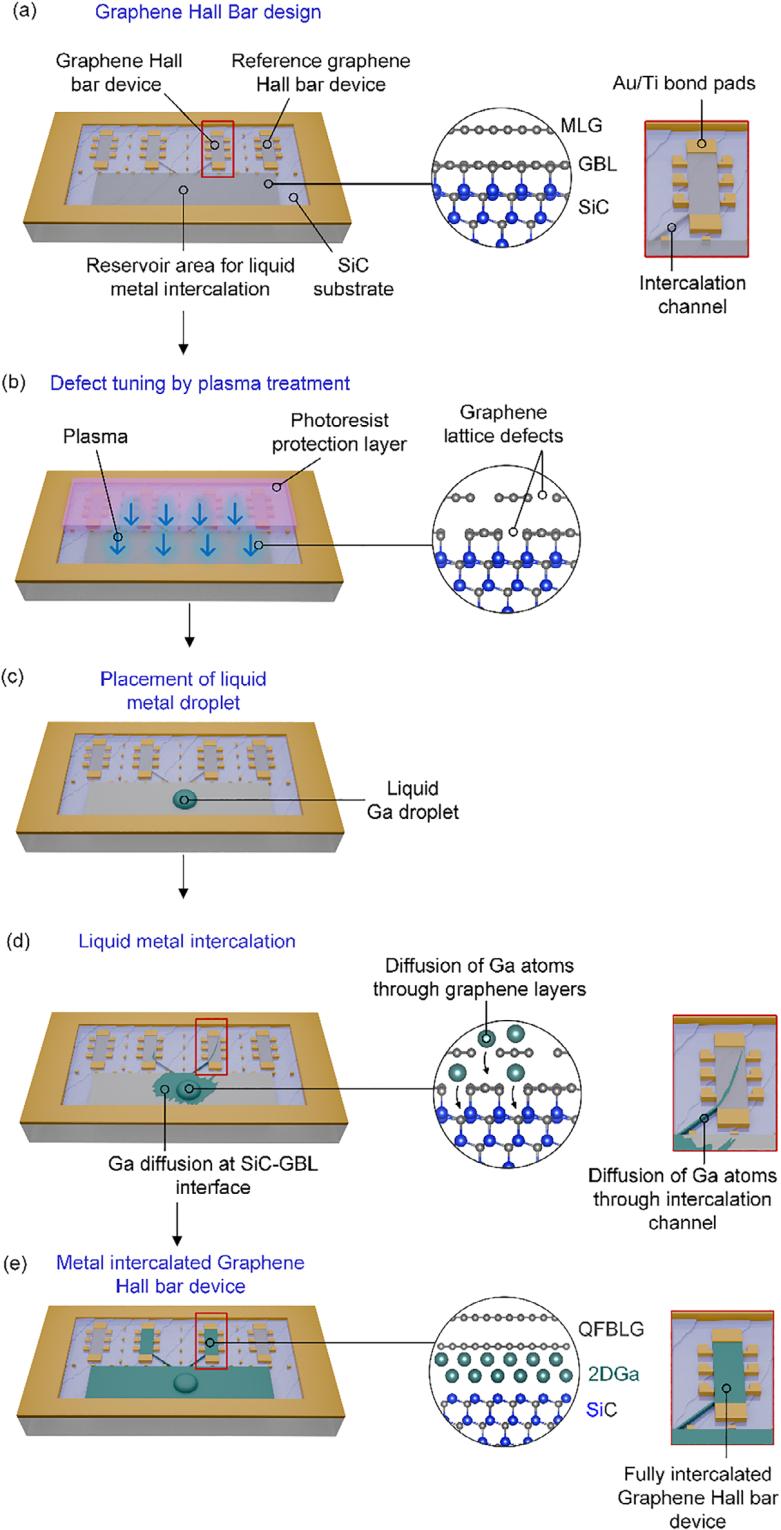
Schematic design of liquid metal intercalation of epitaxial graphene Hall bars. a) Graphene Hall bar design: Schematic representation of the graphene Hall bar device on a SiC substrate, including a reservoir area for liquid metal intercalation and reference Hall bar structures. b) Defect tuning by plasma treatment: controlled plasma treatment applied to selectively introduce lattice defects in the graphene layer. c) placement of liquid metal droplet: a liquid gallium (Ga) droplet is deposited onto the reservoir area, initiating the intercalation process. d) Liquid metal intercalation: Gallium atoms diffuse through the graphene layers and migrate along the SiC‐graphene buffer layer (GBL) interface. e) Formation of the metal‐intercalated graphene Hall bar: intercalated device consisting of decoupled quasi‐freestanding bilayer graphene (QFBLG) and a confined gallium layer (2DGa) between the QFBLG and the SiC substrate.

This method leverages the diffusion properties of the low‐melting‐point metal Ga, enabling the intercalation of epitaxial graphene even at room temperature.^[^ ^18^ ^]^ **Figure** **2**a–e illustrates the key concept of the fabrication process.

The graphene used in this study was synthesized via the polymer‐assisted graphene growth (PASG) method,^[^ ^19^ ^]^ which is specifically designed to minimize undesirable effects such as step bunching of the SiC substrate and to improve graphene quality by introducing polymer molecules as additional carbon sources within epitaxial graphene growth. This optimization enables the formation of an atomically flat epitaxial graphene layer on terraces with heights ranging from 0.50 to 0.75 nm (Figure S3, Supporting Information). By reducing anisotropy in the sheet resistance induced by substrate morphology and miscut angle, PASG enhances the electronic properties of epitaxial graphene.^[^ ^20^ ^]^

The new fabrication approach of the graphene Hall bar design is based on the previously mentioned method using lithography (Figure 1), but starting with epitaxial graphene instead of Ga intercalated QFBLG. However, within the design structure, we incorporated predefined epitaxial graphene intercalation channels and metal reservoir areas that facilitate liquid metal diffusion (Figure 2a). The intercalation channel serves as a connection between the metal reservoir and the graphene Hall bar device, facilitating efficient metal intercalation (Figure 2a). The presence of these dedicated pathways ensures controlled and localized diffusion of the metal intercalant into the graphene Hall bar devices.

Following graphene synthesis, device fabrication was carried out through lithographic structuring to define the Hall bar geometry and intercalation channels. Ensuring a controlled intercalation process requires the precise structuring of a metal reservoir region, which serves as the source for Ga diffusion.

A critical step in the process involved the defect engineering of the metal reservoir region via controlled plasma treatment using a reactive ion etching (RIE) plasma of the exposed epitaxial graphene (Figure 2b), which facilitates subsequent intercalation. During this step, the reservoir area remains uncovered, while the intercalation channels and Hall bars are protected with a photoresist. This selective protection is essential for preserving the intrinsic electronic properties of graphene in the Hall bar, as excessive defect introduction could degrade the performance^[^ ^21^ ^]^ potentially impacting metal‐intercalated graphene systems such as Ga‐intercalated graphene and modifying quantum transport phenomena, including the quantum Hall effect (QHE).

The introduction of additional lattice defects into epitaxial graphene via plasma treatment is a necessary step, as the low intrinsic defect density of as‐grown epitaxial graphene hinders efficient Ga intercalation. Details of the used defect density in epitaxial graphene employed in this method are provided in Figure S4 (Supporting Information).

Following the defect engineering step in epitaxial graphene and the removal of the photoresist protection layer (Figure 2b), a droplet of liquid Ga is placed onto the reservoir area (Figure 2c), where intercalation (Figure 2d) occurs through the controlled application of pressure onto the metal droplet.^[^ ^18^ ^]^ Ga atoms diffuse through lattice defects (Figure 2d) of the monolayer graphene (MLG), migrating towards the interface between the graphene buffer layer (GBL) and the Si‐face of the SiC substrate.^[^ ^18^ ^]^ During this diffusion process, Ga atoms are arranged in a periodicity with respect to Si‐face (0001),^[^ ^14^ ^]^ effectively transforming the epitaxial graphene into quasi‐freestanding bilayer graphene (QFBLG).^[^ ^14^, ^18^ ^]^ We assume that the Ga intercalation into epitaxial graphene is driven by the minimization of interfacial energy. The SiC(0001)/buffer layer interface is energetically unfavorable due to the high lattice strain in the (6√3 × 6√3) R30° reconstruction, partially sp^3^‐hybridized C‐atoms^[^ ^22^ ^]^ and unsaturated Si dangling bonds, which cause a high interfacial energy.

Upon intercalation, the GBL transforms into graphene, relieving strain and restoring complete sp^2^ hybridization. Simultaneously, Ga saturates Si dangling bonds, further stabilizing the interface. This process reduces interfacial energy, thereby facilitating the confinement of metal beneath graphene.

The Ga intercalation propagates from the reservoir area through the intercalation channels into the Hall bars (Figure 2d). The final structure (Figure 2e) represents a fully intercalated graphene Hall bar device. The intercalation propagation strongly depends on the surface topography of the SiC substrate, a factor that will be discussed in detail later.

### 2.2 Implementation and Observation of Metal Intercalation in Epitaxial Graphene Hall Bar Devices

**Figure 3:**
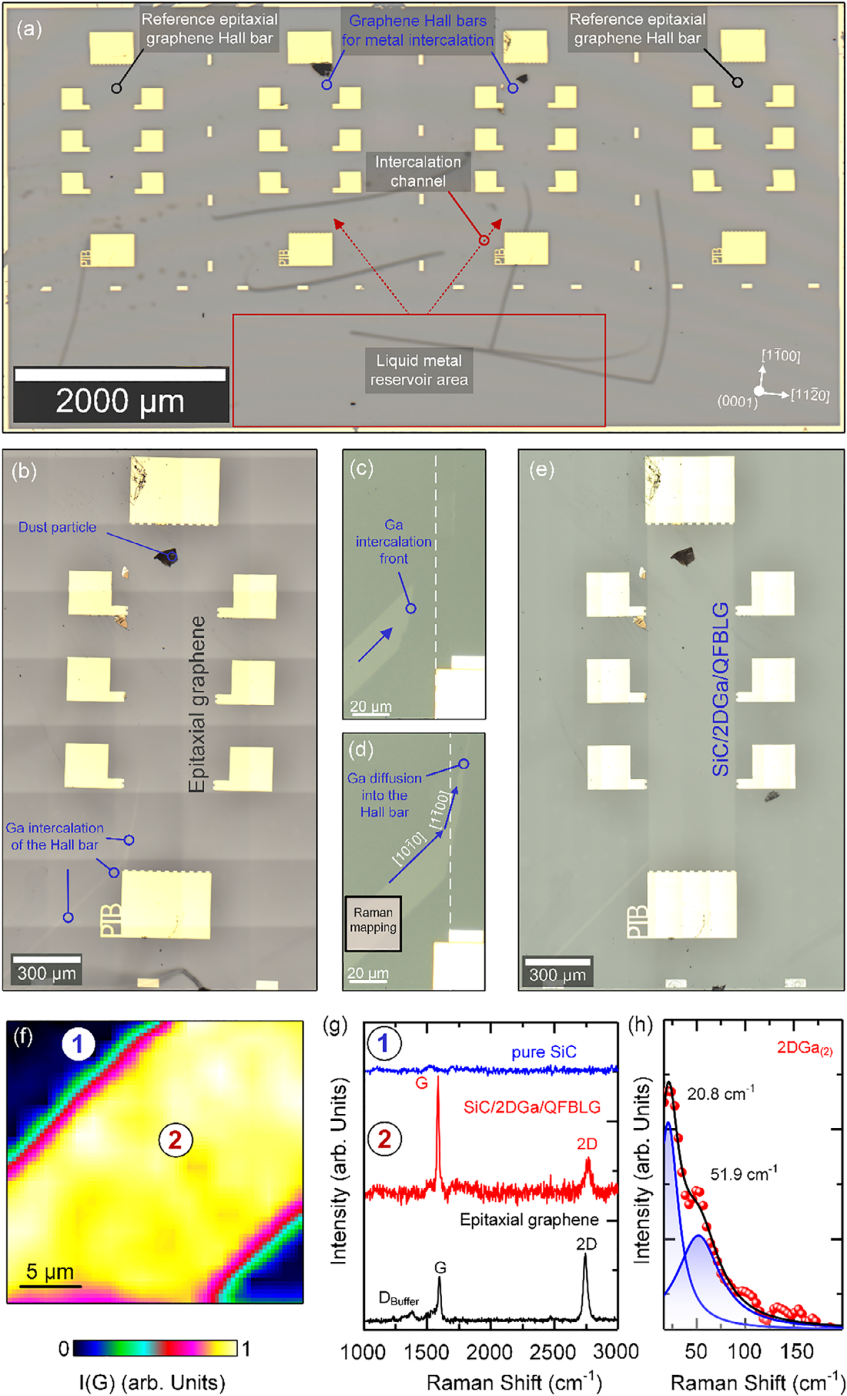
a) Optical microscopy image of the fabricated Hall bar array, showing reference epitaxial graphene Hall bars and Hall bars designed for metal intercalation. The liquid metal reservoir area, where gallium is deposited for intercalation, is outlined in red. Intercalation channels between the reservoir area and the Hall bars are depicted as red arrows. The crystallographic directions of the SiC substrate are indicated on the right lower corner. b–d) Gallium intercalation observed at different time periods e) Fully intercalated Hall bar f) Raman intensity mapping of the G‐band of intercalated QFBLG of the intercalation channel. g) Raman spectra of pure SiC away from the Hall bar device (blue), Ga‐intercalated QFBLG (red), and epitaxial graphene (black) as reference. h) Low‐frequency phonon modes of intercalated Ga layer indicating the 2DGa_(2)_ phase.

**Figure** **3**a shows an optical microscope image of the lithographically fabricated graphene Hall bar structures designed for metal intercalation. The overall arrangement includes reference Hall bars and Hall bars specifically intended for metal intercalation, whereas the reservoir area is highlighted by a red box (Figure 3a). Due to the high optical transparency of graphene, the Hall bars and intercalation channels cannot be clearly distinguished with standard optical microscopy,^[^ ^23^ ^]^ whereas the latter are marked by red arrows (Figure 3a).

The lithographically defined epitaxial graphene Hall bar devices are rotated by a few degrees with respect to the substrate steps along the [$1\bar{1}00]$ crystallographic direction. Each Hall bar device measures approximately (400 × 1600) µm^2^. The reference Hall bars enable a direct comparison with intercalated Hall bars to identify deviations in the quantum Hall effect (QHE) or the emergence of additional electronic phenomena. Note that contamination, such as dust particles, appeared locally (Figure 3b), but did not affect the intercalation process or the magneto‐transport measurements themselves.

The intercalation process has been carried out in a nitrogen atmosphere (at ≈1 bar) to prevent oxidation of the liquid metal. The real‐time progression of Ga intercalation close to room temperature was monitored under an optical microscope, and video recordings of this process are provided in the supplementary materials (Figure S5 and Video SV.1, Supporting Information).

### 2.3 Metal Diffusion Pathways and Kinetics

Figure 3b–e shows microscopy images capturing different stages of the Ga intercalation process over time. The optical contrast of the epitaxial graphene and the intercalated graphene clearly reveals a strong optical contrast difference, which is indicative of Ga incorporation during and after the intercalation.^[^ ^18^ ^]^ Figure 3c already shows a contrast difference, indicating the Ga intercalation towards the Hall bar through the intercalation channel. Figure 3d illustrates the final stages of intercalation, showing Ga approaching the Hall bar and diffusing into it. Unlike other metals, Ga is already in its liquid state close to room temperature, allowing intercalation to proceed even without additional heating. Here, we observed successful intercalation of the reservoir region of approx. 15–20 min (see Video SV.2, Supporting Information). The intercalation dynamics were analyzed by tracking the progression of Ga diffusion over time. Based on the changes observed between Figure 3b,e, the complete intercalation of the Hall bar is estimated to take approximately one day. However, a more precise determination requires further investigation. The migration of Ga atoms from the intercalation channel into the epitaxial Hall bar, spanning a ≈120 µm distance between the intercalation fronts shown in Figure 3c,d, was observed to take ≈7 min. Given that the total length of the intercalation channel is ≈800 µm, we estimate that Ga diffusion through the channel takes ≈47 min.

From these observations, we conclude that the intercalation speed depends on the alignment of the intercalation channel relative to the SiC terraces. As the channel orientation shifts, the dominance of the ($1\bar{1}00$) plane (along the SiC terrace) diminishes, likely due to the increasing number of terrace steps that must be overcome, while the ($10\bar{1}0$) plane serves as the primary diffusion pathway for the metal atoms. The Ga diffusion into adjacent terraces is significantly hindered due to increased potential barriers. Upon entering the Hall bar, the diffusion path of the Ga atoms abruptly changes, realigning along the [$1\bar{1}00$] plane direction, suggesting a substantial impact of the surface topography on the intercalation dynamics.

This directional preference for intercalation suggests that the fabrication process must account for terrace orientation to optimize the homogeneity of intercalation across the Hall bar device. The kinetics of metal diffusion are strongly affected by the surface potential of the SiC terraces. The observed diffusion of Ga at room temperature reveals an anisotropic behavior, predominantly occurring along the $\left[1\bar{1}00\right]$ direction, i.e., along the SiC terraces. This indicates diffusion along the terraces is preferred, guiding the movement of intercalated atoms at the interface between graphene and the SiC. Similar diffusion properties were also observed during the intercalation of lead (Pb) at 320 °C beneath the graphene buffer layer on SiC, demonstrating that this phenomenon is not exclusive to Ga and epitaxial graphene.

The observed diffusion behavior aligns with expectations based on the Ehrlich–Schwoebel barrier,^[^ ^25^ ^]^ which hinders cross‐terrace migration. Thus, we conclude that the observed anisotropic diffusion of Ga atoms is described by a 2D diffusion‐coefficient matrix, reflecting the effective confinement to the surface plane (i.e., the *z*‐direction vanishes).

Specifically,

(1) $$D=\left(\begin{array}{cc} D_{xx} & D_{xy}\\ D_{yx} & D_{yy} \end{array}\right)\;$$

in which *D*_xx_​ and *D*_yy_​ ​ represent the diffusion of Ga atoms along [$1\bar{1}00$] and orthogonal to the SiC terraces (along [$11\bar{2}0$]), respectively. The off‐diagonal components *D*_xy_ and *D*_yx_ represent cross‐ or coupled diffusion processes between the two directions and are neglected for simplicity. Concretely,

(2) $$D_{xx}=\frac{a^{2}}{z}ve^{\left(-\frac{\Delta E_{\left|\right|}}{k_{B}T}\right)}$$

(3) $$D_{yy}=\frac{a^{2}}{z}ve^{\left(-\frac{\Delta E_{⊥}}{k_{B}T}\right)}$$

where *a* is the lattice spacing between neighboring surface sites, *z* is the coordination number (i.e., the number of possible jump directions), *ν* is the attempt frequency for jumps along (perpendicular) the terrace, and Δ*E*_∥_​ and Δ*E*_⊥_​ are the corresponding activation energies. The additional term Δ*E*_ES_​ is the Ehrlich–Schwoebel barrier, which raises the effective activation energy Δ*E*_⊥_ for crossing terrace steps (see Equation 4), resulting in *D*_yy_ < *D*_xx_.

(4) $$\Delta \;E_{⊥}=\Delta E_{\left|\right|}+\Delta E_{\mathrm{ES}}$$

In our model, Δ*E*_⊥_ captures the combined influence of the intrinsic in‐plane diffusion barrier Δ*E*_∥_ and the Ehrlich–Schwoebel barrier Δ*E*_ES_, which elevates the energy required for step‐edge crossing. When Δ*E*_ES_ becomes significant, for instance when the step height of the terrace increases (due to step bunching), cross‐terrace diffusion is strongly suppressed, leading to a dominance of *D*_yy_ in the diffusion matrix (Equation 1). At elevated intercalation temperatures, this anisotropy might diminish. Previous DFT studies on hydrogen diffusion on 3C‐SiC(111) surfaces reported on‐terrace activation barriers of ≈1.15–1.35 eV,^[^ ^26^ ^]^ with substantially higher step‐edge barriers of ≈1.65–1.75 eV, underscoring the role of step‐edge kinetics. Analogous energy barriers are likely to govern atomic motion during Ga intercalation on SiC. A systematic DFT‐based investigation of confined Ga diffusion will be essential to quantify these barriers and elucidate the underlying transport mechanisms. Furthermore, beyond step‐limited kinetics, metal intercalation and the resulting metal diffusion beneath epitaxial graphene are governed by the chemical potential *µ*. In this context, we assume that Fick's law of diffusion is modulated by the chemical potential associated with the incorporation of the metal species, which reflects the lattice energy difference between the highly strained graphene buffer layer and the strain‐relaxed QFBLG. The buffer layer in epitaxial graphene is subject to significant lattice strain due to the covalent bonding with the underlying Si face of the SiC substrate. Upon intercalation, this strain is partially relieved, releasing lattice energy and thereby reducing the total potential energy of the system. More importantly, DFT studies of dysprosium (Dy) adsorption on epitaxial graphene have demonstrated that the chemical potential critically influences both the preferred adsorption sites of metal atoms on graphene and the resulting surface reconstructions of the metal layer.^[^ ^27^ ^]^ Similarly, DFT investigations of lead (Pb) intercalation in graphene on SiC reveal that metal coverage, in conjunction with intrinsic strain within the intercalated layer, governs phase stability.^[^ ^28^ ^]^ These competing surface reconstructions, ranging from amorphous‐like to close‐packed configurations, are separated by small energy differences, underscoring the sensitivity of interfacial structure to chemical potential.

Raman mapping was performed to investigate the intercalation channels after Ga diffusion (Figure 3d – black box, f). Figure 3f presents the lateral intensity distribution of the G peak across the mapped area, clearly reflecting the shape of the intercalation channel. Raman spectra collected (Figure 3g, blue and red spectra) from two different regions are marked in Figure 3f. The black Raman spectrum in Figure 3g represents epitaxial graphene before Ga intercalation, measured on the reservoir area.

Each Raman spectrum within the acquired Raman mapping (Figure 3f) was subtracted by a reference spectrum of bare SiC to reveal the G peak of graphene that is otherwise superimposed by the second‐order SiC bands between 1450 and 1950 cm^−1^ (Figure S6, Supporting Information). The blue‐colored Raman spectrum (Figure 3g) represents the subtracted Raman signal outside of the intercalation channel (Figure 3f, No. 1), confirming the complete removal of epitaxial graphene after the etching process used for Hall bar lithography.

In general, a subtracted Raman spectrum of epitaxial graphene before intercalation (Figure 3g, black spectrum) exhibits characteristic G and 2D peaks^[^ ^29^ ^]^ at 1602 and 2761 cm^−1^ associated with strained monolayer graphene, along with broad and flattened phonon bands from the graphene buffer layer (see *D*_Buffer_ phonon mode between 1200 and 1500 cm^−1^, Figure 3g black spectrum) in the spectral range of 1200 to 1600 cm^−1^.^[^ ^30^ ^]^ Following Ga intercalation, the spectral background of the graphene buffer layer disappears, as indicated by the red spectrum in Figure 3g, accompanied by an increase in intensity of the G peak and a spectral downshift from 1602 to approximately 1586 cm^−1^, indicating a strain release due to lattice relaxation of graphene after the Ga intercalation. Furthermore, the intensity and peak shape of the 2D peak undergo notable changes, reflecting the decoupling of the graphene buffer layer and formation of QFBLG.^[^ ^14^, ^31^ ^]^ The observed decrease in the 2D peak intensity is attributed to strong doping effects resulting from charge transfer between QFBLG and the intercalated Ga layers. Furthermore, no *D* peak around 1350 cm^−1^ is observed (Figure 3g, red spectrum), indicating the absence of lattice defects in QFBLG and confirming that the structure of the graphene lattice is preserved after the liquid‐metal intercalation.

Two characteristic low‐energy phonon modes of the Ga layer appear near the Rayleigh line at approximately 20.8 and 51.9 cm^−1^ (Figure 3h) within the Raman measurements of the intercalation channel, indicating a so‐called 2DGa_(2)_ phase.^[^ ^32^ ^]^ Wetherington et al. showed that Ga intercalated at the epitaxial graphene/SiC interface segregates into two structurally distinct 2D phases: a 2D‐Ga_(1)_ with Raman features at 28, 43, and 109 cm^−1^, and a 2D‐Ga_(2)_ marked by the pronounced low‐frequency phonon modes at approximately 26 and 54 cm^−1^. Although low‐frequency Raman modes in layered systems are typically attributed to rigid interlayer‐shear modes,^[^ ^33^ ^]^ the observed Raman active phonons in 2D‐Ga_(1)_ and 2D‐Ga_(2)_ are not attributed to these.^[^ ^33^ ^]^ Rather, it is proposed that the observed phonon modes reflect a structural transition that occurs after the 2D Ga layers exceed a critical thickness or concentration related to an intralayer‐packing rearrangement.^[^ ^33^ ^]^

Within Raman measurements of both the intercalation channel and the Hall bar devices (Figure S7, Supporting Information), only the 2D‐Ga_(2)_ phase has been observed, indicating that a critical layer thickness of Ga has already been exceeded.^[^ ^33^ ^]^ However, as detailed below, SEM measurements reveal localized contrast variations indicative of spatial fluctuations in Ga layer thickness. This suggests that the number of Ga layers may not be uniform across the sample.

Scanning‐tunnelling microscopy (STM) was used to quantify the Ga layer thickness beneath the QFBLG. Measurements were taken on a reference SiC/2D‐Ga/QFBLG sample fabricated with the identical defect‐engineering and Ga‐intercalation protocol employed for the Hall bar devices. Height profiles recorded across the abrupt interface between pristine epitaxial graphene and the intercalated region (Figure S8, Supporting Information) show a step height of approximately 0.42 nm, indicating a two‐atomic‐layer Ga film, which seems to be fully consistent with STEM data reported in the literature.^[^ ^14^ ^]^ We note, however, that the Ga thickness is likely to vary locally across the Hall‐bar and along the intercalation channels.

Outside the intercalation channel, the Raman mapping reveals no detectable Ga signatures by investigating the Ga intensity peak, indicating that no deintercalation of Ga has occurred by accumulation of liquid Ga along the edges of the intercalation channel (Figure S7, Supporting Information). This observation underpins the stability of the intercalated Ga layer confined between QFBLG and the SiC substrate after the intercalation process.

### 2.4 Spectroscopic Characterization of Ga‐Intercalated QFBLG

Throughout this study, spectroscopic measurements using SPA‐LEED, ARPES, XPS, and four‐point probe STM were performed on Ga‐intercalated reference samples prepared with the same defect‐engineering and Ga‐intercalation protocol as the devices. This strategy avoids any device modification by sample transport, vacuum annealing in analysis systems, or exposure to ionizing radiation during XPS.

The Ga intercalation of epitaxial graphene has been further validated through ARPES, SPA‐LEED, and XPS measurements, complementing Raman spectroscopy. These measurements were conducted on large Ga‐intercalated epitaxial graphene samples without structured Hall bars, where the sample quality is expected to be comparable.

**Figure 4:**
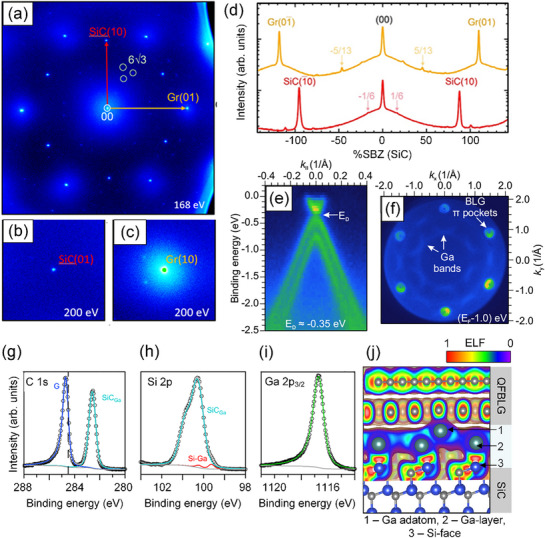
a) SPA‐LEED image of the Ga intercalated graphene on SiC acquired at 168 eV, showing the first‐order SiC and Gr diffraction spots. b,c) Zoom in around the SiC and Gr spots at 200 eV. d) High‐resolution spot profiles along the SiC (red) and Gr (yellow) direction. The curves are offset in *y*‐axis for better visibility. e) Electronic band structure along E‐k dispersion measured at the K‐point, perpendicular to the Γ‐K direction. f) Fermi surface map showing the emergence of Ga‐derived bands and bilayer graphene (BLG) pockets, confirming the impact of intercalation on the electronic structure. g) C 1s core level spectrum of intercalated QFBLG h) Si 2p spectrum revealing Si‐Ga bond feature i) Ga 2p_3/2​_ spectrum, confirming the presence of Ga in the graphene intercalated areas. j) DFT calculated Electron Localization Function (ELF). ELF mapping shows the electronic charge distribution in QFBLG with intercalated gallium. The first intercalated Ga layer exhibits strong localization near the Si‐face, forming a covalently polarized bond, whereas in between the gallium layer and the QFBLG the additional adatom shows more delocalized metallic behavior. The color scale indicates the degree of electron localization, where red represents highly localized electrons and blue indicates delocalized states.

**Figure** **4**a shows a high‐resolution SPA‐LEED image recorded on a SiC/2DGa/QFBLG sample. The bright Gr spots indicate the successful Ga intercalation. Notably, the Gr spots, as well as the (00) spot, are accompanied by a coherent background, as clearly seen in Figure 4c ‐ the so‐called bell‐shaped component, which is a hallmark of free‐standing graphene.^[^ ^34^ ^]^

Moreover, the faint remnants of the (6 × 6) and 6√3 spots (also see Figure 4c,d) suggest the decoupling of the majority of the buffer layer, leading to the formation of QFBLG. However, the absence of Ga‐induced superstructures might indicate a (1 × 1) saturation of the interface (or Ga termination of the Si bonds), that has also been observed for other metal‐intercalated graphene systems.^[^ ^35^ ^]^ The electronic interaction of QFBLG and the confined Ga layers was investigated by ARPES measurements. The ARPES data were also acquired on a reference SiC/2DGa/QFBLG sample prepared with the same defect‐engineering and Ga‐intercalation protocol that was applied to the Hall‐bar devices. Figure 4e presents the *E–k* dispersion of QFBLG after Ga‐intercalation around the K̄ point of graphene's Brillouin zone, where the characteristic π bands for bilayer graphene are visible. The observed splitting of the π bands originates from the interlayer interaction between the two graphene layers.^[^ ^36^ ^]^ Notably, the downward shift of the Dirac point to *E*_D_ ≈ 350 meV indicates negative charge carrier doping, which results from charge transfer between the confined 2D Ga layer and the two graphene layers above. The intercalated Ga layer at the interface serves as an electron donor, effectively introducing n‐type doping into QFBLG.

The Fermi surface in Figure 4f illustrates the first Brillouin zone of bilayer graphene, dominated by the characteristic electron pockets at the high‐symmetry K and K' points. Additionally, a weak background signal around Γ reveals the presence of Ga‐derived bands, which are assigned as near‐free‐electron‐like states, which disperse upward towards the K and K’ points of graphene.^[^ ^14^ ^]^ However, these states do not directly hybridize with the Dirac states of graphene. Instead, the observed shift of the Dirac point confirms an electron transfer from the confined 2D Ga layer to the graphene layers, resulting in n‐type doping.^[^ ^14^ ^]^

Figure 4g–i illustrates representative XP spectra of a SiC/2DGa/QFBLG sample. The C 1s signal is composed of two components, corresponding to carbon atoms that are bound either in the QFBLG (G) or the SiC bulk below the Ga layer (SiC_Ga_). The binding energy of the bulk signal (*E*_B_ =  282.6 eV) exhibits a notable shift of 1.1 eV to a lower binding energy compared to that observed for non‐intercalated epitaxial graphene.^[^ ^37^ ^]^ This can be attributed to an altered surface band bending, which occurs concurrently with the rearrangement of the surface bonding configuration. This phenomenon has been observed on numerous occasions in the context of metal intercalation of epitaxial graphene layers.^[^ ^24^ ^]^

A similar shift is evident in the bulk component of the Si 2p spectrum depicted in Figure 4h (SiC_Ga_ at *E*_B_ (Si 2p_3/2_) =  100.3 eV), which originates from silicon atoms bound within the bulk. Furthermore, an additional component, Si─Ga, was included in the analysis to reproduce the shoulder observed at low binding energy (*E*_B_ =  99.6 eV), which can be assigned to the topmost Si atoms bound to the intercalated Ga layer. It is noteworthy that the surface component is considerably sharper than the corresponding bulk component. This phenomenon has been previously observed for Si─Pb bonds.^[^ ^24^ ^]^ It is postulated that this is due to an enhanced screening of the core hole for bonding to the metallic Ga. Figure 4i depicts the Ga 2p_3/2_ spectrum, which exhibits a single asymmetric component at 1116.6 eV, in good agreement with the reported value for elemental Ga (*E*_B_ = 1116.67 eV).^[^ ^38^ ^]^ We note that the absence of oxide signals underlines the protective effect of the graphene layers in the case of this sample. Furthermore, no signal from pristine epitaxial graphene is detected in the Si 2p and C 1s spectra (Figure 4g,h), indicating that the entire XPS measurement area is homogeneously intercalated.

To resolve the interfacial bonding states in SiC/2DGa/QFBLG, we conducted electron localization function (ELF) calculations using DFT. The supercell consists of a Si‐terminated 6H‐SiC (0001) substrate (Figure 4j, No. 3) and a single intercalated monolayer of Ga atoms (Figure 4j, No. 2) confined between the substrate and the QFBLG. To probe the vertical bonding evolution within the Ga monolayer, a single Ga adatom (Figure 4j, No. 1) was placed atop the intercalated layer, serving as a minimal perturbation to isolate and visualize the local electronic interaction between adjacent Ga atoms under confinement.

The ELF color scale indicates the degree of electron localization, where red regions represent highly localized electrons and blue areas indicate delocalized states.

The ELF distribution (Figure 4j) reveals distinct bonding regimes across the interface. Between the first Ga layer (Figure 4j, No. 2) and the Si‐terminated surface (Figure 4j, No. 3), the ELF values exceed 0.75 (marked as red regions), indicating a high degree of electron localization. This suggests the formation of polarized, partially covalent bonds between Ga and Si atoms. In contrast, the region between the Ga adatom (Figure 4j, No. 1) and the underlying Ga monolayer shows significantly decreased ELF < 0.5 (blue regions), characteristic of delocalized electronic states, indicating a weak, predominantly metallic interaction between the Ga adatom and the intercalated Ga layer, lacking significant directional bonding. Thus, the results reveal a layered electronic asymmetry induced by confinement, suggesting that the first Ga layer is structurally and electronically pinned to the Si face, while additional Ga atoms above it remain metallic.

Bader analysis^[^ ^39^ ^]^ was carried out to evaluate charge transfer between the confined Ga layer and QFBLG by calculating the net charge density *n*_Layer_ within the atomic layers in the supercell obtained from the charge density of DFT calculations. Here, the atomic net charges *q*_net_ of the Bader analysis were summed for each atomic layer (QFBLG, 2D‐Ga monolayer, Si‐face, etc.) and divided by the in‐plane supercell area (Figure S9, Supporting Information). A detailed description of the calculation of net charge density as well as the averaged atomic net charges $\bar{q}$_net_ within a layer is listed in Table S1 (Supporting Information).

As shown in Figure S9 (Supporting Information), the Bader analysis indicates that Ga induces a net charge density of *n*_Layer_ ≈ 4.5 × 10^13^ cm^−2^ ($\bar{q}$_net_ ≈−0.014*e* per atom) in the lower and approx. 6.8 × 10^12^ cm^−2^ (−0.002*e* per atom) in the upper graphene layer, which yields a total net charge of approx. 5.2 × 10^13^ cm^−2^ of electrons transferred into QFBLG. The Bader analysis indicates a stronger interfacial coupling of Ga to the adjacent graphene layer, which might result in a charge‐doping gradient in QFBLG (Figure S9, Supporting Information). A weak bond polarization of the covalent Ga─Si bond at the Si face, as seen in the ELF, is indicated by the averaged Bader net charges. The 2D Ga layer averages 0.078*e* per atom, indicating donor character also towards the Si‐face. The Si‐face averages 1.945*e* per atom, slightly lower than deeper Si layers at 2.644*e* per atom (Figure S9, Supporting Information). Furthermore, the alternating averaged net charge of Carbon atoms (−2.429*e* per atom) and Si atoms (2.644*e* per atom) reveals the intrinsic Si─C polarization along SiC (0001). Compared to ARPES (*E*_D_ ≈ 0.35 eV and a resulting electron concentration of *n*_e,ARPES_ ≈ 8.12 × 10^12^ cm^−2^), the calculated net charge density might be overestimated. The methodology of extracting *n*_e,ARPES_ is described in detail later. This difference may reflect residual uncertainties in the DFT charge density for very large supercells, where practical limits on k‐point sampling and plane‐wave cutoffs can modestly influence the results. Additionally, the apparent mismatch might occur because *n*_e,ARPES_ probes electronic band filling via photoemission, whereas the Bader analysis quantifies the atomic net charge within the Bader volume.

### 2.5 Magnetotransport Measurements of Ga‐Intercalated QFBLG

Ga intercalation and diffusion beneath graphene is a dynamic process that leads to spatial variations in the number and arrangement of Ga layers at the QFBLG/Si‐face interface. This behavior reflects the energetic stability of Ga mono‐, bi‐, and trilayer under confinement, with growth strongly influenced by the substrate's surface topography, particularly the SiC terrace edges.^[^ ^14^ ^]^

**Figure 5:**
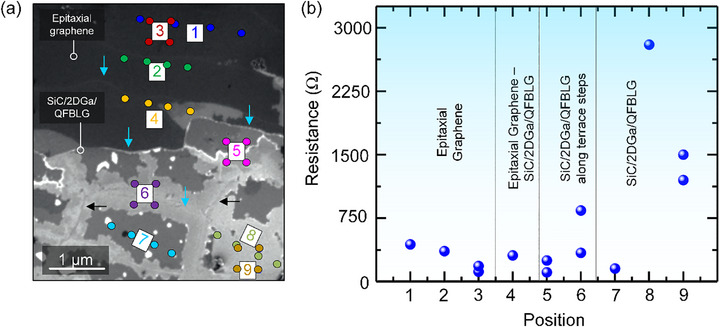
a) Scanning electron microscopy (SEM) image of the sample with color‐coded tip arrangements realized for transport measurements. Resistance measurements were performed in both linear and square configurations at different locations: epitaxial graphene (positions 1–3), the transition region between epitaxial graphene and intercalated SiC/2DGa/QFBLG (position 4), and Ga‐intercalated graphene (positions 5–9). The observed contrast variations in the intercalated regions may be attributed to differences in the number of intercalated gallium layers. Terrace steps on 4H‐SiC are marked with blue arrows b) Position‐dependent resistance extracted from four‐point probe STM measurements.

**Figure** **5**a presents an SEM image of the intercalation front between epitaxial graphene and SiC/2DGa/QFBLG, which was measured on large Ga‐intercalated epitaxial graphene samples. Here, the non‐intercalated epitaxial graphene area exhibits a homogeneous dark contrast in the upper part (Figure 5a, top region), whereas the intercalated region (Figure 5a, bottom region) shows pronounced contrast variations. Similar observations have been reported, where the number of intercalated Ga layers was quantified through correlation analysis in SEM imaging.^[^ ^40^ ^]^ Thus, the SEM contrast in our measurements suggests strong dependence of the Ga interface layer on substrate steps (mostly horizontal—Figure 5a, marked by blue arrows) as well as graphene nanowrinkles (vertically connecting steps—Figure 5a, black arrows).

Local sheet resistance was investigated using four‐point probe STM transport measurement in both linear and square tip configurations on different sample areas (Figure 5a,b).^[^ ^41^ ^]^ Uniform sheet resistance was observed across epitaxial graphene (positions 1–4) and at the phase boundary along SiC terraces with a resistance ranging between 0.9 and 2.3 kΩ sq^−1^. However, at terrace edges (marked with blue arrows, Figure 5a), the resistance increased significantly, reaching values between 2.3 and nearly 14 kΩ sq^−1^ (Figure 5b, positions 6, 8, and 9). A comparable sheet resistance to epitaxial graphene (position 2) was observed within SiC/2DGa/QFBLG on a terrace (position 7).

Notably, the high pressure of the STM probe tips may locally modify the intercalated Ga layer or introduce defects, potentially affecting the measured resistance. Nevertheless, these findings indicate that terrace edges can act as scattering centers for charge carriers in metal‐intercalated layers, with possible contributions from variations in layer thickness or intrinsic lattice defects. Since no insulating areas were observed, the macroscopic transport behavior of the device (across several hundred µm) will likely experience an average effect of interface variations.

**Figure 6:**
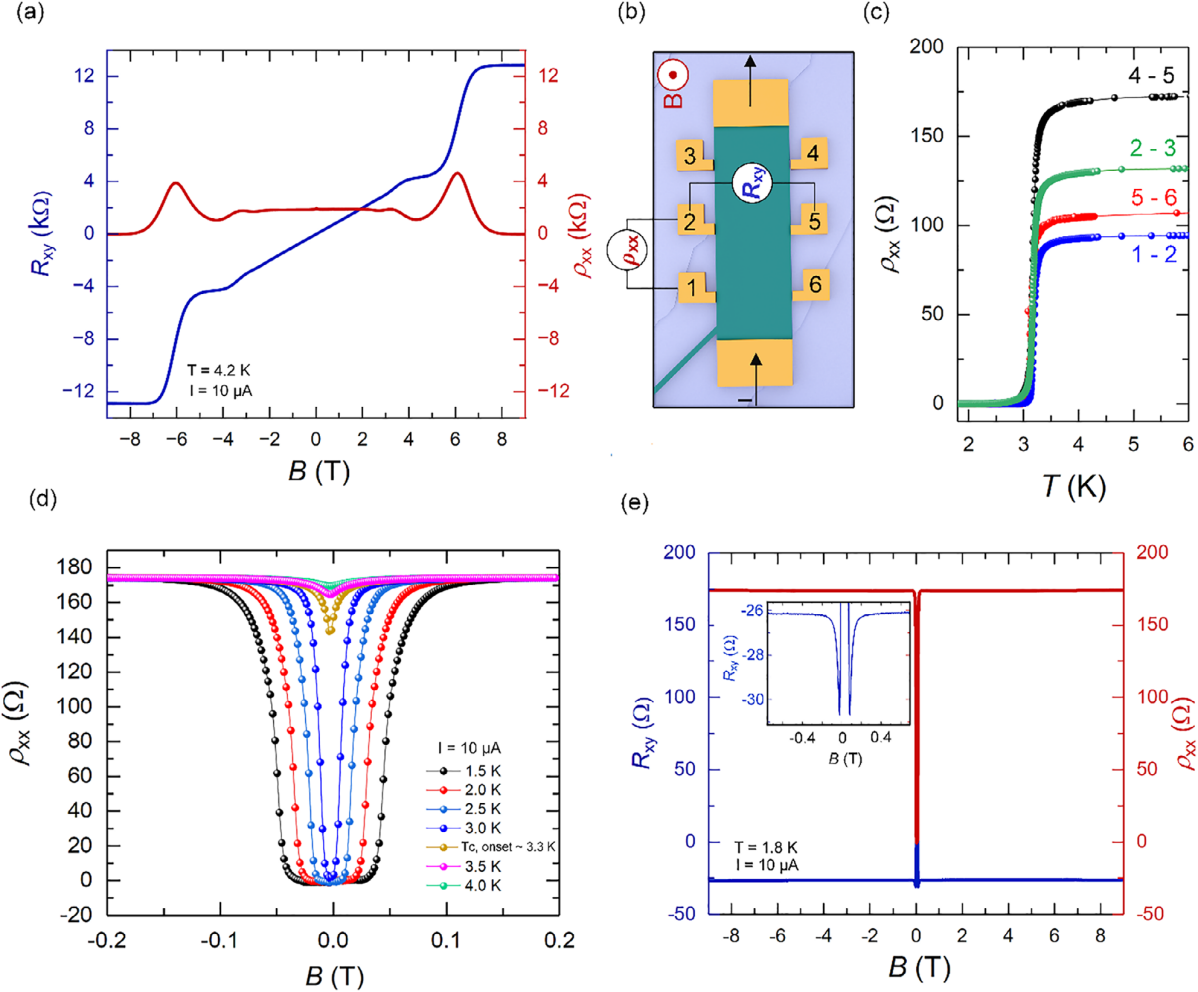
a) Quantum Hall effect of epitaxial graphene at *T* = 4.2 K and *I* = 10 µA. Blue (red) ordinate depicts the Hall resistance (longitudinal resistivity). b) Schematic of the measurement setup for longitudinal (*ρ*_xx_) and Hall (*R*_xy_) resistances under an external magnetic field B. c) Temperature‐dependent longitudinal resistance (ρ_xx_) of Ga‐intercalated quasi‐freestanding bilayer graphene (SiC/2DGa/QFBLG). The superconducting phase in gallium of SiC/2DGa/QFBLG is observed at *T*_c,onset_ ≈ 3.5 K. d) Temperature‐dependent sweep of magnetic field strength to investigate the change of *T*_c_. e) Sweep of magnetic field strength in Ga‐intercalated QFBLG at 1.8 K and *I* = 10 µA. Blue (red) ordinate depicts the Hall resistance (longitudinal resistivity).

The electronic properties of epitaxial graphene and Ga‐intercalated QFBLG were investigated using standard low‐temperature 4‐point magnetotransport measurements, with the intercalation channel physically interrupted (cut off) to eliminate potential parasitic electronic effects. The Hall resistance curves *R*_xy_ (blue curve) and the longitudinal resistivity *ρ*_xx_ (red curve) of the devices are shown for epitaxial graphene (**Figure** **6**a), and for the Ga‐intercalated QFBLG Hall bar (Figure 6c–e). The wiring diagram setup for *ρ*_xx_ and *R*_xy_ measurements is shown schematically in Figure 6b.

The results on the epitaxial graphene sample at 4.2 K, and using a current (*I*) of 10 µA, show typical QHE features (Figure 6a) with fully developed quantum Hall resistance plateaus, and a value for *R*_xy_ ≈ 12.9 kΩ for filling factor *ν* = 2 at high magnetic fields (*B* ≥ 7 T). In turn, the longitudinal resistivity *ρ*_xx_ vanishes in the same region as expected for edge channels.^[^ ^42^ ^]^

Temperature‐dependent measurements of the longitudinal resistivity *ρ*_xx_(T) in zero magnetic field, in the large Ga‐intercalated QFBLG Hall bar, using the same current of *I* = 10 µA, show superconductivity (Figure 6c). Four distinct *ρ*_xx_(*T*) measurements are displayed, for four different contact pairs of similar separation (Figure 6b,c), collinear with the current. The observed superconducting onset temperature $T_{c}^{\mathrm{onset}}$ ≈ 3.5 K is approximately 0.5 K lower than the values reported earlier in the literature, while the zero‐resistance transition temperature $T_{c}^{0}$ ≈ 3.0 – 3.25 K is in good agreement.^[^ ^14^ ^]^ A detailed description of the occurrence of superconductivity in the confined Ga layer can be found elsewhere.^[^ ^14^ ^]^

It has been reported that $T_{c}^{\mathrm{onset}}$ is influenced by surface topography, with large step bunching slightly suppressing $T_{c}^{\mathrm{onset}}$.^[^ ^14^ ^]^ They also reported that the slope of *R*(T) varies with current direction, showing different behavior parallel and perpendicular to step edges in small‐ and large‐step samples. In our study, however, such step directional effects of the SiC substrate cannot be resolved, as the Hall bar orientation deviates slightly from the [$1\bar{1}00]$ crystallographic direction of 4H‐SiC, averaging out anisotropies. Therefore, we attribute the dominant impact of surface topography on *ρ*_xx_ and *R*_xy_ to inhomogeneities in the Ga layer distribution as observed in SEM (Figure 5a).

The variation of *ρ*_xx_(T, H) with applied magnetic field *B* of increasing strength shows that superconductivity is suppressed, and the superconducting transition width broadens (Figure 6d). The critical magnetic field, *B*_c2_ ≈ 100 mT, observed in the macroscopic Hall bar device (Figure 6d), is comparable to the 130 mT reported by Briggs et al.^[^ ^14^ ^]^ implying a similar superconducting coherence length. Our intercalated Ga films display a critical current *I*_c_ ≈ 200 µA (Figure S10, Supporting Information), justifying the use of currents in the 10 µA range for the characterization and measurements.

Figure 6e shows the longitudinal (*ρ*_xx_, red) and transverse (*R*_xy_, blue) resistance as a function of the applied magnetic field in the Ga‐intercalated QFBLG system. As the magnetic field increases beyond 100 mT, superconductivity is suppressed, leading to the emergence of electrical resistance in both *ρ*_xx_ and *R*_xy_. However, rather than showing QHE characteristics, *ρ*_xx_(H) remains almost constant with magnetic field to 9 T. *R*_xy_(H), on the other hand, exhibits pronounced peak anomalies at *B* = *B*_c2_ = 100 mT (see Figure 6d inset), followed by almost constant negative resistance at 26 Ω to 9 T. This seemingly anomalous result is likely a consequence of inhomogeneous current paths leading to current jetting effects that result in admixing of *R*_xy_ and *ρ*_xx_ channels (effectively equivalent to misaligned transverse contact pads).

*R*_xy_(H) in the Ga intercalated sample is found to contain both symmetric and antisymmetric in‐field components (Figure S11, Supporting Information). We attribute the symmetric‐in‐field component to non‐uniform currents in the Ga film, and an antisymmetric‐in‐field component characteristic of the conventional Hall effect. The antisymmetric component of *R*_xy_ implies (assuming single‐band conduction, reasonable for 1–3 layer Ga) an estimated carrier density of SiC/2DGa/QFBLG in excess of *n*_e, Hall_ ≈10^15^ cm^−2^, which is two orders of magnitude higher than for as‐grown epitaxial graphene (typically *n*_e, Hall_ ≈ 1 × 10^13^ cm^−2^).^[^ ^43^ ^]^

In contrast, ARPES measurements reveal that the Dirac point is shifted to *E*_D_ ≈ 0.35 eV below the Fermi level (*E*_F_). At this doping level, only a single π* pocket is occupied at *E*_F_ (see Figure 4e). Applying Luttinger's theorem,^[^ ^44^, ^45^ ^]^ the corresponding electron density in the QFBLG is estimated to be *n*_e,ARPES_ ≈ 8.12 × 10^12^ cm^−2^. Here, *n*_e,ARPES_ was estimated by assuming a circular π* pocket and neglecting a trigonal warping effects, as the doping level of the QFBLG is relatively low.

It should be noted as mentioned before that *n*_e,ARPES_ reflects the electronic band filling via photoemission, whereas magnetotransport measurements probe mobile charge carriers contributing to electrical conduction. However, in this context, the exceptionally high carrier concentration observed in magnetotransport measurements cannot be solely attributed to charge accumulation in the QFBLG. Such an extreme confinement of free carriers exclusively within the 2DGa film is physically unlikely without introducing substantial effects into QFBLG, as observed in heavily doped QFMLG using gadolinium as intercalant, resulting in a high electron carrier concentration of 9.5 × 10^14^ cm^−2^ up to VHS, resulting in flat bands and polaron bands.^[^ ^8^ ^]^ This has not been observed in our ARPES measurements of SiC/2DGa/QFBLG.

Anomalous transverse resistance effects at *B* = *B*_c2_ have been observed before in a number of superconducting systems, which may arise from distinct underlying mechanisms.

Prior studies have found that inhomogeneous superconductors can exhibit anomalous behavior due to variations in the longitudinal and Hall resistivity with temperature and magnetic field, leading to an even‐in‐field transverse response.^[^ ^46^ ^]^ Furthermore, electronic inhomogeneities in superconducting thin films can induce a highly non‐uniform current distribution, giving rise to transverse resistance at macroscopic length scales, even in structurally uniform samples.^[^ ^47^ ^]^ Additionally, anomalous transverse resistance has been also observed in the topological superconductor β‐Bi_2_Pd and attributed to broken interfacial inversion symmetry, potentially linked to topological surface states.^[^ ^48^ ^]^ Furthermore, it cannot be excluded that local structural phase transitions occur in the Ga layers at low temperatures, affecting the Hall resistance.

The observed behavior in the Ga‐intercalated QFBLG system may result from a combination of these effects, as spatial inhomogeneities in the intercalated Ga layers have been observed in our SEM measurements (Figure 5a). However, further investigations, including additional transport measurements and theoretical modeling, are currently being planned to deconvolute these contributions and elucidate the dominant mechanism. Indeed, systematic investigations including spatially resolved transport measurements and microscopic mapping of intercalation homogeneity will be crucial to fully elucidate the interplay between structural inhomogeneity and electronic transport in SiC/2DGa/QFBLG devices.

Overall, our approach demonstrates that the proposed lithographic method for epitaxial graphene, combined with post‐lithography intercalation via specifically designed intercalation channels, enables the scalable fabrication of well‐defined intercalated Hall bar devices. These devices are suitable for systematic magneto‐transport investigations and offer a platform for exploring the electronic properties of confined 2D metal layers.

## 3 Conclusion

We have successfully developed a lithographically controlled intercalation approach based on liquid metal intercalation for fabricating metal‐intercalated graphene Hall bar devices. This method enables precise control over intercalation dynamics while preserving the integrity of confined metal layers. By integrating lithographic structuring with post‐lithography intercalation through dedicated diffusion channels, we achieved scalable and reproducible device fabrication, overcoming processing‐induced deintercalation and ensuring structural stability. The introduction of engineered intercalation channels is crucial for directing the diffusion of Ga, allowing for localized and controlled metal incorporation into defect‐free Hall bar devices.

Magnetotransport measurements confirm superconductivity in Ga‐intercalated quasi‐freestanding bilayer graphene, with a superconducting transition at $T_{c}^{\mathrm{onset}}$ ≈ 3.5 K. The significantly higher charge carrier concentration in 2DGa, which is several orders of magnitude above that of graphene, indicates that the superconducting path is primarily located within the 2D Ga layer. Furthermore, we observe the absence of a quantized Hall effect, while the transverse resistance exhibits both symmetric and antisymmetric field components. We attribute the symmetric‐in‐field component to non‐uniform currents within the Ga film, whereas the antisymmetric component reflects the conventional Hall effect.

The ability to precisely modulate metal intercalation at the nanoscale establishes a robust platform for tunable intercalated graphene systems, offering new opportunities for proximity‐induced superconductivity, quantum transport studies, and electronic applications. Given its scalability and compatibility with existing fabrication techniques, this method could be extended to metal‐intercalated graphene‐based electronic devices, paving the way for the integration of functional quantum materials into next‐generation superconducting and nanoelectronic circuits.

## 4 Experimental Section

### Polymer‐Assisted Graphene Growth (PASG)

Monolayer epitaxial graphene was grown on semi‐insulating 4H‐SiC (0001) substrates (10 × 5 mm^2^) using the so‐called polymer‐assisted sublimation growth (PASG) method. The SiC wafer from II‐VI Comp. has a nominal miscut of about 0.04°. The graphene samples were prepared using the polymer‐assisted sublimation growth (PASG) technique, which involves polymer adsorbates formed on the 4H‐SiC surface through liquid‐phase deposition from a solution of photoresist (AZ5214E) in isopropanol, followed by sonication and brief rinsing with isopropanol. The graphene layer growth was processed at 1750 °C (argon atmosphere, approx. 1 bar, 6 min, with zero argon flow) with pre‐vacuum annealing at 900 °C.

### Defect Engineering and Gallium‐Intercalation of Epitaxial Graphene Using LiMIT

Defect engineering of the reservoir area on the epitaxial graphene sample was performed using reactive ion etching (RIE). Detailed information on the adjustment of defect density in graphene, as determined by Raman spectroscopy, is presented in Figure S2 (see Supporting Information). The commercial gallium (99.99% purity) was purchased from Heraeus. The liquid metal intercalation technique (LiMIT) was employed, involving the placement of a Ga droplet on the metal reservoir area of the graphene Hall bar device. The sample was gently heated to 70 °C in a nitrogen atmosphere to decrease the surface tension of the metal droplet and to improve wetting on the graphene surface, while an external pressure was applied locally to the liquid metal droplet for 10 min.

### Confocal Raman Spectroscopy

Confocal Raman spectroscopy was performed using a Witec Alpha 300 RA equipped with a 300 grooves per mm grating, a Nd:YAG laser with an excitation wavelength of 488 nm (2.54 eV), and a 600 mm focal length. Raman mapping was conducted over a (20 × 20) µm^2^ area with a step resolution of 0.2 µm. To prevent laser‐induced damage or heating effects, the laser power was maintained at a level below 2 mW. The system is equipped with a volume Bragg grating for ultra‐low frequency which enables the observation of low‐frequency phonon modes of intercalated Ga.

### Angle‐Resolved Photoelectron Spectroscopy (ARPES)

ARPES measurements were performed with a NanoESCA (Scienta Omicron) using He I radiation (21.2 eV). The samples were degassed at around 150 °C prior to measurements.

### X‐Ray Photoelectron Spectroscopy (XPS)

XPS measurements were conducted using Al Kα radiation (h*v* = 1486.6 eV) from a Specs XR50 X‐ray source monochromatized with a Specs Focus 500 monochromator. The samples were degassed at around 150 °C before measurements. All XPS measurements were taken at room temperature with a base pressure better than 3 × 10^−10^ mbar and all the core level data were taken at *E*_Pass_ = 10 eV.

### Scanning Electron Microscopy (SEM) and Four‐Point Probe Scanning Tunneling Microscopy (4pp‐STM) Measurements

SEM and 4pp‐STM measurements were performed in UHV at a base pressure of 5 × 10^−10^ mbar. SEM images were acquired at 15 kV and 1 nA. Without annealing, the investigated area was cleaned by removing adsorbates during continuous scanning with a STM tip. Transport measurements were performed by four electrochemically etched tungsten tips. The probes were brought into the tunneling regime by feedback‐controlled approaching. The ohmic contact was established by manually approaching each tip with feedback control switched off. For both linear and square tip arrangements, a tip spacing of 400 nm was realized (700 nm in case of the first measurements) and a source current ranging from ‐1…+1 µA applied.

### Magnetotransport Measurement

The magnetotransport measurements were performed in a commercial Oxford Instruments bath cryostat with a 12 T superconducting magnet, an ADRET Electronique current source, and an HP 3458 multimeter.

### Density Functional Theory (DFT)

The structural relaxations and electronic structure calculations were performed using the Quantum ESPRESSO package within the plane‐wave pseudopotential approach. The lattice relaxation and subsequent calculation of the electron localization function (ELF) were performed using an expanded supercell based on the (√3 × √3)R30° supercell of SiC (0001). The lateral supercell size is approx. (13.84 ×15.97) Å^2^ with a height of approx. 16 Å and a vacuum space of approx. 12 Å. The constructed cell consists of QFBLG, a Ga monolayer (2DGa), and a single Ga adatom, forming the (SiC/2DGa monolayer/Ga adatom/QFBLG) system on the (0001) surface (Figure S1, Supporting Information). The SiC surface consists of two SiC layers, with the bottom layer passivated by hydrogen atoms along (0$00\bar{1})$.

The large supercell size has been used to avoid interaction of the Ga adatom with the next cell. Furthermore, the lattice relaxation was carried out in multiple steps. Initially, during the relaxation of the (SiC/2DGa monolayer/QFBLG) system, all atoms below the Si‐face were kept fixed, allowing only the Si atoms of the Si‐face, the Ga monolayer, and the QFBLG layer to relax. The Ga atoms of the Ga monolayer were positioned in a (1 × 1) arrangement relative to the Si‐face.

Following this relaxation step, a Ga adatom was placed in a hollow site position on the Ga monolayer, situated at the Ga monolayer/QFBLG interface, and the system was subsequently relaxed again.

The Perdew‐Burke‐Ernzerhof (PBE) parametrization^[^ ^49^ ^]^ of the generalized gradient approximation (GGA‐PBE) was used for the exchange‐correlation functional. Furthermore, projector augmented wave (PAW) pseudopotentials were employed as well as van der Waals correction using Grimme‐D2.^[^ ^50^ ^]^ The lattice relaxation was performed using a plane‐wave energy cutoff of 816 eV and a force convergence threshold of 0.003 eV Å^−1^. Details of Bader analysis are described in the Supporting Information.

## Conflict of Interest

The authors declare no conflict of interest.

## Supporting information



Supporting Information

Video 1

Video 2

## Data Availability

The data that support the findings of this study are available from the corresponding author upon reasonable request.

## References

[1] M. Bora , P. Deb , JPhys Mater. 2021, 4, 034014.

[2] Z. Mamiyev , C. Tegenkamp , 2D Mater. 2024, 11, 025013.

[3] Y. Cao , V. Fatemi , S. Fang , K. Watanabe , T. Taniguchi , E. Kaxiras , P. Jarillo‐Herrero , Nature 2018, 556, 43. 10.1038/nature26160. 29512651

[4] A. K. Geim , K. S. Novoselov , Nat. Mater. 2007, 6, 183. 10.1038/nmat1849. 17330084

[5] E. Mazaleyrat , S. Vlaic , A. Artaud , L. Magaud , T. Vincent , A. Gómez‐Herrero , S. Lisi , P. Singh , N. Bendiab , V. Guisset , P. David , S. Pons , D. Roditchev , C. Chapelier , J. Coraux , 2D Mater. 2020, 8, 015002.

[6] C. Li , Y.‐F. Zhao , A. Vera , O. Lesser , H. Yi , S. Kumari , Z. Yan , C. Dong , T. Bowen , K. Wang , H. Wang , J. L. Thompson , K. Watanabe , T. Taniguchi , D. Reifsnyder Hickey , Y. Oreg , J. A. Robinson , C.‐Z. Chang , J. Zhu , Nat. Mater. 2023, 22, 570. 10.1038/s41563-023-01478-4. 36781950

[7] K. H. Kim , H. He , C. Struzzi , A. Zakharov , C. E. Giusca , A. Tzalenchuk , Y. W. Park , R. Yakimova , S. Kubatkin , S. Lara‐Avila , Phys. Rev. B 2020, 102, 165403. 10.1103/PhysRevB.102.165403.

[8] S. Link , S. Forti , A. Stöhr , K. Küster , M. Rösner , D. Hirschmeier , C. Chen , J. Avila , M. C. Asensio , A. A. Zakharov , T. O. Wehling , A. I. Lichtenstein , M. I. Katsnelson , U. Starke , Phys. Rev. B 2019, 100, 121407. 10.1103/PhysRevB.100.121407.

[9] P. Rosenzweig , H. Karakachian , S. Link , K. Küster , U. Starke , Phys. Rev. B 2019, 100, 035445. 10.1103/PhysRevB.100.035445. 33156643

[10] P. Rosenzweig , U. Starke , Phys. Rev. B 2020, 101, 201407. 10.1103/PhysRevB.101.201407. 33156643

[11] L. Daukiya , M. N. Nair , S. Hajjar‐Garreau , F. Vonau , D. Aubel , J. L. Bubendorff , M. Cranney , E. Denys , A. Florentin , G. Reiter , L. Simon , Phys. Rev. B 2018, 97, 035309. 10.1103/PhysRevB.97.035309.

[12] M. Einenkel , K. B. Efetov , Phys. Rev. B 2011, 84, 214508. 10.1103/PhysRevB.84.214508.

[13] S. A. Herrera , G. Parra‐Martínez , P. Rosenzweig , B. Matta , C. M. Polley , K. Küster , U. Starke , F. Guinea , J. Á. Silva‐Guillén , G. G. Naumis , P. A. Pantaleón , ACS Nano 2024, 18, 34842. 10.1021/acsnano.4c12532. 39652458

[14] N. Briggs , B. Bersch , Y. Wang , J. Jiang , R. J. Koch , N. Nayir , K. Wang , M. Kolmer , W. Ko , A. De La Fuente Duran , S. Subramanian , C. Dong , J. Shallenberger , M. Fu , Q. Zou , Y.‐W. Chuang , Z. Gai , A.‐P. Li , A. Bostwick , C. Jozwiak , C.‐Z. Chang , E. Rotenberg , J. Zhu , A. C. T. van Duin , V. Crespi , J. A. Robinson , Nat. Mater. 2020, 19, 637. 10.1038/s41563-020-0631-x. 32157191

[15] Y. Yin , M. Kruskopf , P. Gournay , B. Rolland , M. Götz , E. Pesel , T. Tschirner , D. Momeni , A. Chatterjee , F. Hohls , K. Pierz , H. Scherer , R. J. Haug , H. W. Schumacher , Phys. Rev. Appl. 2025, 23, 014025. 10.1103/PhysRevApplied.23.014025.

[16] W. Poirier , S. Djordjevic , F. Schopfer , O. Thévenot , C. R. Phys. 2019, 20, 92. 10.1016/j.crhy.2019.02.003.

[17] S. Wundrack , T. Tschirner , K. Pierz , A. P. Bakin , Patent application German Patent and Trade Mark Office 2025, 102025107546.8.

[18] S. Wundrack , D. Momeni , W. Dempwolf , N. Schmidt , K. Pierz , L. Michaliszyn , H. Spende , A. Schmidt , H. W. Schumacher , R. Stosch , A. Bakin , Phys. Rev. Mater. 2021, 5, 024006. 10.1103/PhysRevMaterials.5.024006.

[19] M. Kruskopf , D. M. Pakdehi , K. Pierz , S. Wundrack , R. Stosch , T. Dziomba , M. Götz , J. Baringhaus , J. Aprojanz , C. Tegenkamp , J. Lidzba , T. Seyller , F. Hohls , F. Ahlers , H. W. Schumacher , 2D Mater. 2016, 3, 15172720.

[20] D. Momeni Pakdehi , J. Aprojanz , A. Sinterhauf , K. Pierz , M. Kruskopf , P. Willke , J. Baringhaus , J. P. Stöckmann , G. A. Traeger , F. Hohls , C. Tegenkamp , M. Wenderoth , F. J. Ahlers , H. W. Schumacher , ACS Appl. Mater. Interfaces 2018, 10, 6039. 10.1021/acsami.7b18641. 29377673

[21] P. Kot , J. Parnell , S. Habibian , C. Straßer , P. M. Ostrovsky , C. R. Ast , Phys. Rev. B 2020, 101, 235116. 10.1103/PhysRevB.101.235116.

[22] T. Schumann , M. Dubslaff , M. H. Oliveira , M. Hanke , J. M. J. Lopes , H. Riechert , Phys. Rev. B 2014, 90, 041403. 10.1103/PhysRevB.90.041403.

[23] P. Blake , E. W. Hill , A. H. Castro Neto , K. S. Novoselov , D. Jiang , R. Yang , T. J. Booth , A. K. Geim , Appl. Phys. Lett. 2007, 91, 063124 10.1063/1.2768624.

[24] F. Schölzel , P. Richter , A. D. P. Unigarro , S. Wolff , H. Schwarz , A. Schütze , N. Rösch , S. Gemming , T. Seyller , P. Schädlich , Small Struct. 2025, 6, 2400338. 10.1002/sstr.202400338.

[25] F. Rabbering , H. Wormeester , F. Everts , B. Poelsema , Phys. Rev. B 2009, 79, 075402. 10.1103/PhysRevB.79.075402.

[26] K. Seino , A. Oshiyama , Appl. Surf. Sci. 2021, 561, 149927. 10.1016/j.apsusc.2021.149927.

[27] Y. Han , J. W. Evans , M. C. Tringides , Phys. Rev. Mater. 2021, 5, 074004. 10.1103/PhysRevMaterials.5.074004

[28] Y. Han , M. Kolmer , J. W. Evans , M. C. Tringides , Phys. Rev. Mater. 2024, 8, 044002. 10.1103/PhysRevMaterials.8.044002

[29] D. S. Lee , C. Riedl , B. Krauss , K. von Klitzing , U. Starke , J. H. Smet , Nano Lett. 2008, 8, 4320. 10.1021/nl802156w. 19368003

[30] F. Fromm , M. H. Oliveira Jr , A. Molina‐Sánchez , M. Hundhausen , J. M. J. Lopes , H. Riechert , L. Wirtz , T. Seyller , New J. Phys. 2013, 15, 043031, 10.1088/1367-2630/15/4/043031.

[31] F. Speck , M. Ostler , J. Röhrl , J. Jobst , D. Waldmann , M. Hundhausen , L. Ley , H. B. Weber , T. Seyller , Mater. Sci. Forum 2010, 645–648, 629. 10.4028/www.scientific.net/MSF.645-648.629.

[32] W. He , M. T. Wetherington , K. A. Ulman , J. L. Gray , J. A. Robinson , S. Y. Quek , J. Phys. Chem. Lett. 2022, 13, 4015. 10.1021/acs.jpclett.2c00719. 35485838

[33] M. Wetherington , F. Turker , T. Bowen , A. Vera , S. Rajabpour , N. Briggs , S. Subramanian , 2D Mater. 2019, 31, 041003. 10.1088/2053-1583/ac2245

[34] Z. Mamiyev , C. Tegenkamp , Surf. Interfaces 2022, 34, 102304. 10.1016/j.surfin.2022.102304.

[35] Z. Mamiyev , N. O. Balayeva , C. Ghosal , D. R. T. Zahn , C. Tegenkamp , Carbon 2025, 234, 120002. 10.1016/j.carbon.2025.120002.

[36] T. Ohta , A. Bostwick , T. Seyller , K. Horn , E. Rotenberg , Science 2006, 313, 951. 10.1126/science.1130681. 16917057

[37] K. V. Emtsev , F. Speck , T. Seyller , L. Ley , J. D. Riley , Phys. Rev. B 2008, 77, 155303. 10.1103/PhysRevB.77.155303.

[38] S. Evans , Surf. Interface Anal. 1985, 7, 299. 10.1002/sia.740070609.

[39] W. Tang , E. Sanville , G. Henkelman , J. Phys.: Condens. Matter 2009, 21, 084204. 10.1088/0953-8984/21/8/084204. 21817356

[40] H. El‐Sherif , N. Briggs , B. Bersch , M. Pan , M. Hamidinejad , S. Rajabpour , T. Filleter , K. W. Kim , J. Robinson , N. D. Bassim , ACS Appl. Mater. Interfaces 2021, 13, 55428. 10.1021/acsami.1c14091. 34780159

[41] I. Miccoli , F. Edler , H. Pfnür , C. Tegenkamp , J. Phys.: Condens. Matter 2015, 27, 223201. 10.1088/0953-8984/27/22/223201. 25985184

[42] Y. Yin , M. Kruskopf , S. Bauer , T. Tschirner , K. Pierz , F. Hohls , R. J. Haug , H. W. Schumacher , Appl. Phys. Lett. 2024, 125, 064001. 10.1063/5.0223723.

[43] C. Coletti , C. Riedl , D. S. Lee , B. Krauss , L. Patthey , K. von Klitzing , J. H. Smet , U. Starke , Phys. Rev. B 2010, 81, 235401. 10.1103/PhysRevB.81.235401.

[44] J. M. Luttinger , J. C. Ward , Phys. Rev. 1960, 118, 1417. 10.1103/PhysRev.118.1417.

[45] J. M. Luttinger , Phys. Rev. 1960, 119, 1153. 10.1103/PhysRev.119.1153.

[46] A. Segal , M. Karpovski , A. Gerber , Phys. Rev. B 2011, 83, 094531. 10.1103/PhysRevB.83.094531.

[47] S. Sengupta , A. Farhadizadeh , J. Youssef , S. Loucif , F. Pallier , L. Dumoulin , K. Saha , S. Pujari , M. Óden , C. Marrache‐Kikuchi , M. Monteverde , Phys. Rev. B. 2005, 112, 014514. 10.48550/arXiv.2407.16662.

[48] X. Xu , Y. Li , C. L. Chien , Nat. Commun. 2022, 13, 5321. 10.1038/s41467-022-32877-x 36085297 PMC9463149

[49] J. P. Perdew , K. Burke , M. Ernzerhof , Phys. Rev. Lett. 1996, 77, 3865. 10.1103/PhysRevLett.77.3865. 10062328

[50] S. Grimme , J. Comput. Chem. 2006, 27, 1787. 10.1002/jcc.20495. 16955487

