# Supplementary material for: Lithographically Controlled Liquid Metal Diffusion in Graphene: Fabrication and Magnetotransport Signatures of Superconductivity
Source: Adv Mater. 2025 Oct 30;38(5):e11992. doi: 10.1002/adma.202511992 (PMC12822529; doi:10.1002/adma.202511992)
Supplement: Supplementary file 1 — Supporting Information [file ADMA-38-e11992-s001.docx]

**Supplementary information:** Lithographically-controlled liquid metal diffusion in graphene: Fabrication and magneto-transport signatures of superconductivity

S. Wundrack^1,2,3^, M. Bothe^1,3^, M. Jaime^1^, K. Küster^5^, M. Gruschwitz^4^, Y. Yin^1^, Z. Mamiyev^4^, P. Schädlich^4^, B. Matta^5^, S. Datta^5^, M. Eckert^1,2^, C. Tegenkamp^4^, U. Starke^5^, R. Stosch^1^, H.W. Schumacher^1^, T. Seyller^4^, K. Pierz^1^, T. Tschirner^1^, A. Bakin^2,3^

^1^ Physikalisch-Technische Bundesanstalt, Bundesallee 100, 38116 Braunschweig, Germany

^2^ Institut für Halbleitertechnik, Technische Universität Braunschweig, Hans-Sommer Straße 66, D-38106 Braunschweig, Germany

^3^ Laboratory of Emerging Nanometrology (LENA) der Technischen Universität Braunschweig, Langer Kamp 6 a/b, 38106 Braunschweig, Germany

^4^ Institut für Physik, Technische Universität Chemnitz, Reichenhainer Str. 70, 09126 Chemnitz, Germany

^5^ Max-Planck-Institut für Festkörperforschung, Heisenbergstraße 1, 70569 Stuttgart, Germany

**This supplementary information presents:**

1. Supercell configuration used in DFT calculations **(Fig. S1)**
2. Potassium iodide exposure of Ga-intercalated QFBLG **(Fig. S2)**
3. AFM measurements of PASG epitaxial graphene samples **(Fig. S3)**
4. Defect tuning of epitaxial graphene **(Fig. S4)**
5. Monitoring of Ga-diffusion beneath QFBLG **(Fig. S5, video V1 & V2)**
6. Raman spectrum of Ga-intercalated graphene and non-intercalated epitaxial graphene **(Fig. S6)**
7. Raman mapping of 2DGa_(2)_ phase across intercalaton channel and Hall bar device **(Fig. S7)**
8. Scanning tunneling microscopy for determination of Ga layer thickness across the epitaxial graphene-SiC/2DGa/QFBLG transition zone **(Fig. S8)**
9. Bader analysis of SiC/2DGa/QFBLG **(Fig. 9)**
10. Critical current *I*c measured in superconducting SiC/2DGa/QBFLG Hall bar **(Fig. S10)**
11. Magneto-Transport: *R*_xy_(H) in SiC/2DGa/QFBLG sample containin symmetric and antisymmetric in-field components **(Fig. S11)**
12. **Supercell configuration used in DFT calculations**


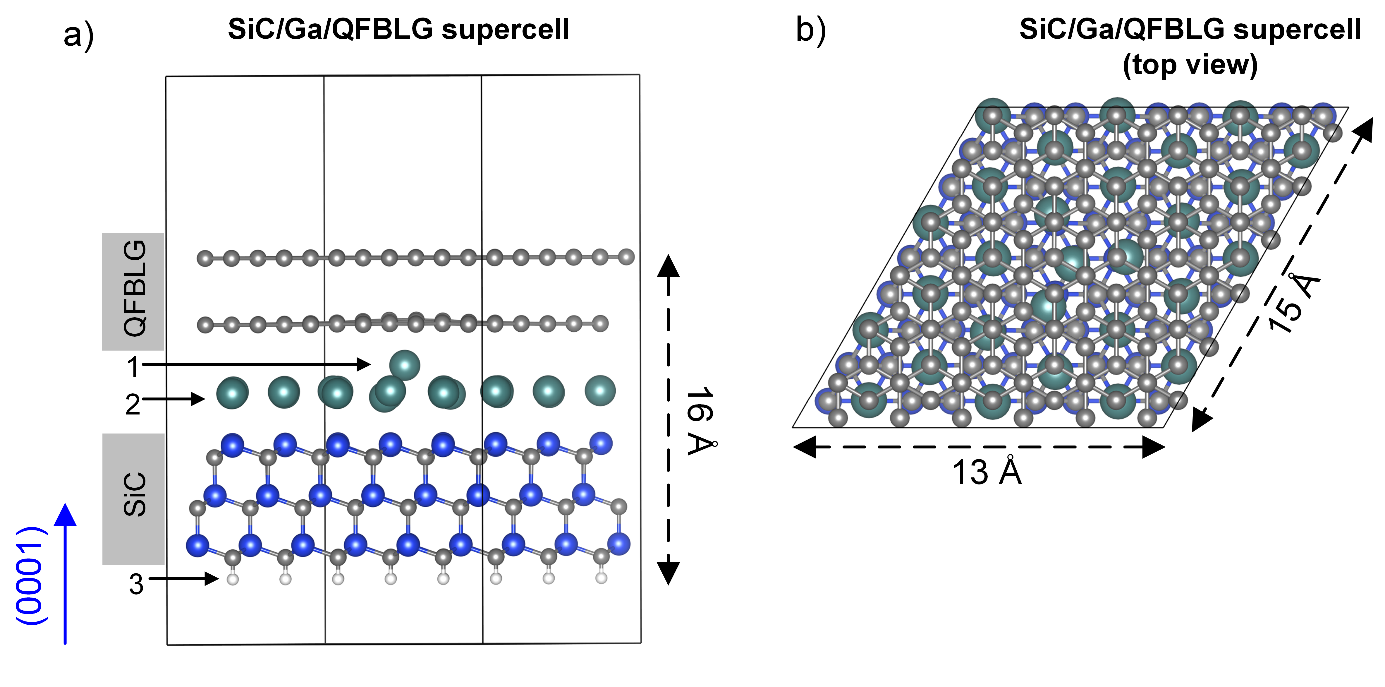


15.97 Å

13.84 Å

**Fig S.1 a)** Expanded supercell of Si/Ga/QFBLG based on the (√3 × √3)R30° supercell of SiC (0001) consisting of 361 atoms. The supercell area is approx. (13.84 × 15.97) Å² with a height of approx. 16 Å and a vacuum space of approx. 12 Å. The constructed cell consists of QFBLG in AB-stacking configuration, a Ga monolayer (No. 2), and a single Ga adatom (No. 1) on the SiC (0001) surface. The SiC surface consists of two SiC layers, with the bottom layer passivated by hydrogen atoms (No. 3) along (0$00\bar{1})$. **b)** Top view of the Si/Ga/QFBLG supercell.

1. **Potassium iodide exposure of Ga-intercalated QFBLG**


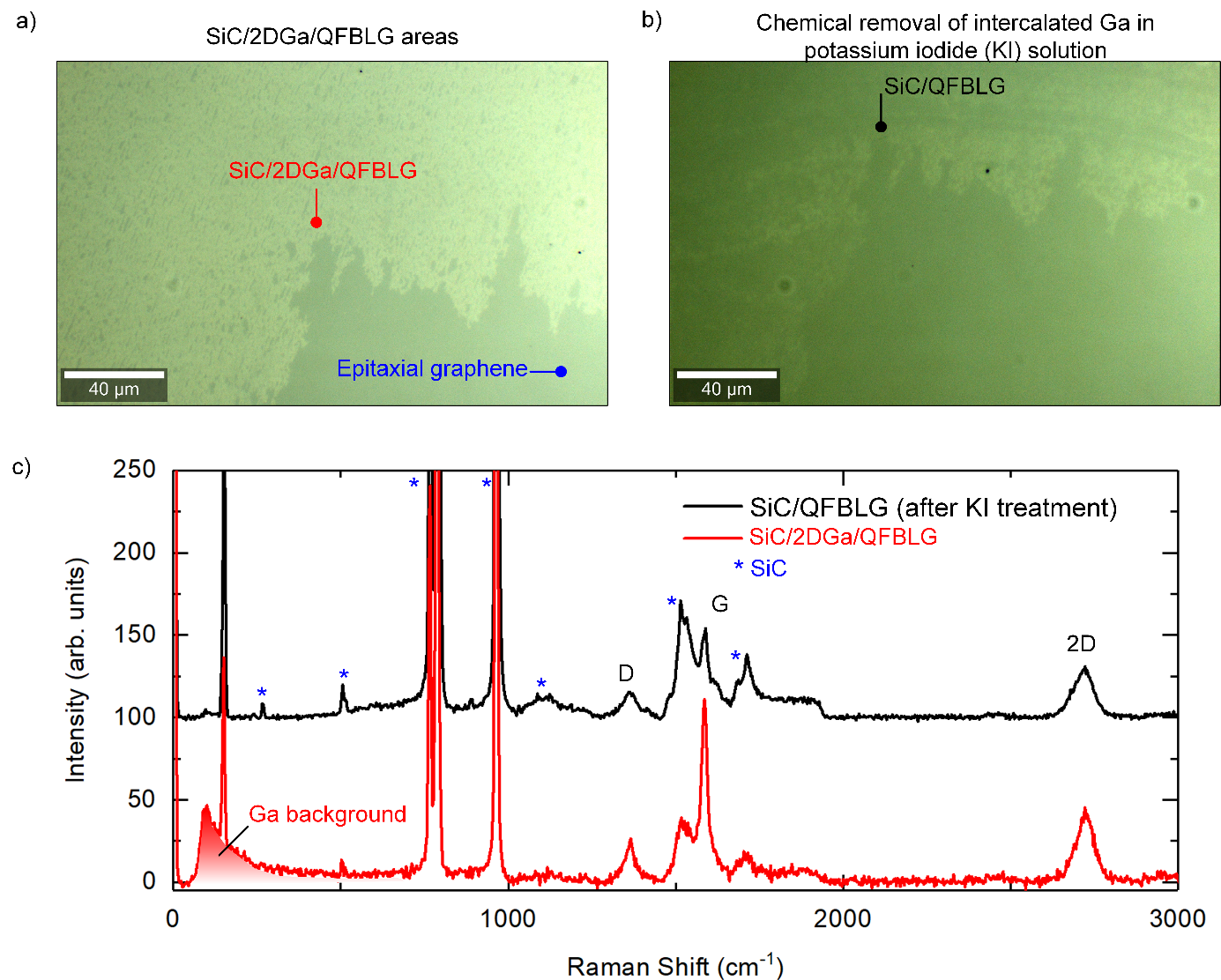


**Fig S.2** **a–b)** Optical microscopy images of a SiC/Ga/QFBLG reference sample before and after potassium iodide (KI) exposure. The sample was prepared with the same defect-engineering and Ga-intercalation protocol as the devices. **a)** Bright regions correspond to areas of SiC/2DGa/QFBLG, while dark regions indicate regions of epitaxial graphene. **b)** The same area after KI treatment shows a pronounced contrast change. This suggests that the intercalated Ga layer has been largely removed. **c)** Raman spectra acquired from the SiC/2DGa/QFBLG regions before (red spectrum) and after (black spectrum) KI exposure. The characteristic graphene fingerprint peaks D, G, and 2D peak are present in both spectra, indicating that the graphene lattice remains largely intact after treatment. The D peak, which originates from lattice defects and is attributed to prior plasma exposure, shows no significant change in intensity, suggesting no substantial alteration in defect density. Significant spectral changes appear in the low-wavenumber region. In the red spectrum (before KI exposure), a broad background is observed between 0 and 500 cm⁻¹, which is attributed to the presence of intercalated Ga. The characteristic Ga phonon modes at 21 and 54 cm⁻¹ are not detected due to the use of an edge filter instead of a Bragg filter, which blocks Raman shifts below 70 cm⁻¹. In contrast, the black spectrum (after KI exposure) reveals the absence of the Ga-related background in the same region, strongly indicating that Ga has been effectively removed by KI treatment. Further investigations are necessary to gain deeper insight into the underlying mechanisms responsible for Ga removal upon KI exposure.

1. **AFM measurements of PASG epitaxial graphene samples**
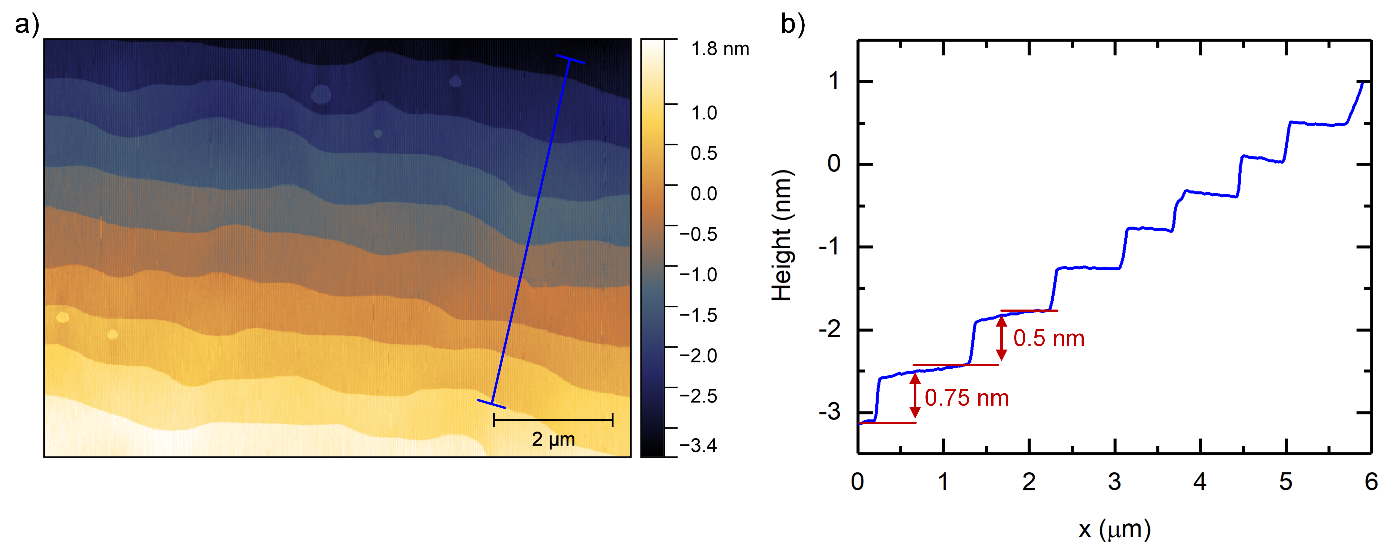


**Fig S.3.** AFM measurements on PASG epitaxial graphene samples. a) depcits well ordered terraces steps on SiC (0001) b) with a homogenous step height between 0.5 and 0.75 nm revealing supressed step bunching. Terrace size varies between 1000 nm down to 500 nm

1. **Defect tuning of epitaxial graphene**


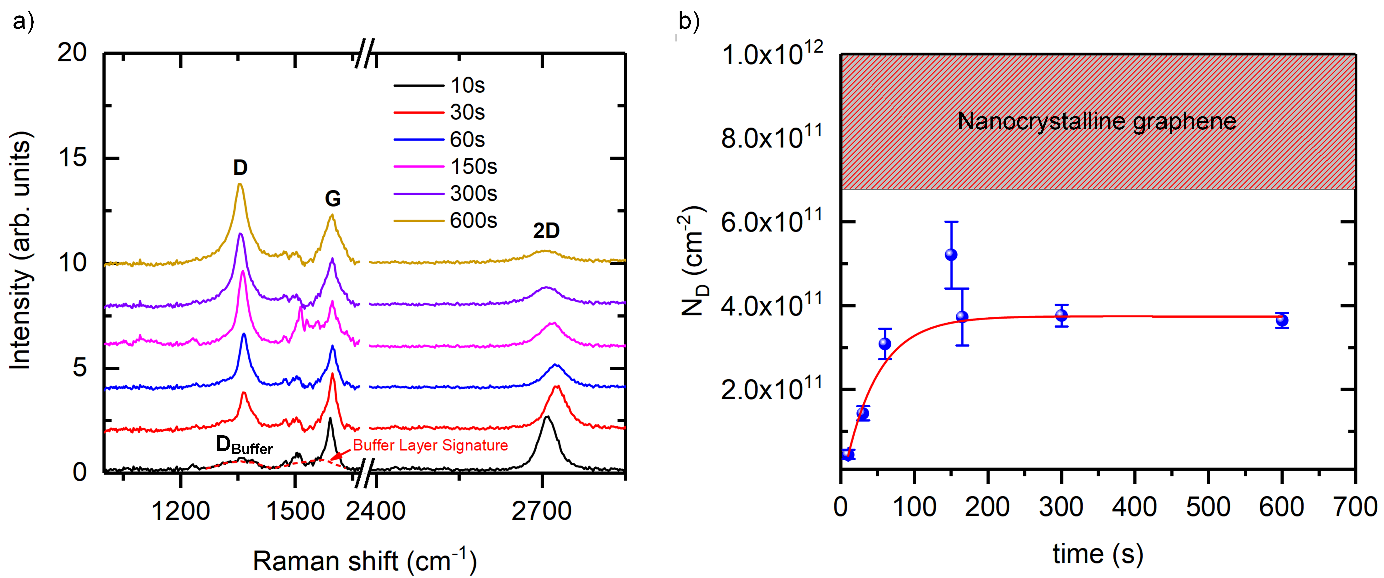


**Fig S.4.** Optimization of defect density in epitaxial graphene via remote plasma treatment in an atomic layer deposition (ALD) system for Ga intercalation **a)** Raman spectra of an epitaxial graphene sample subjected to remote plasma exposure for varying durations. The black spectrum represents pristine epitaxial graphene, exhibiting characteristic features of the graphene buffer layer (e.g., D_Buffer_), as well as the G and 2D peaks of the graphene layer. With increasing plasma exposure time, a progressive increase in the D peak intensity and a corresponding decrease in the 2D peak intensity are observed, indicating a gradual introduction of lattice defects. **b)** Defect density (N_D_) estimated from the D/G intensity ratio. An initial increase in defect density is observed, rising from approximately 1 × 10¹¹ cm⁻² to 4 × 10¹¹ cm⁻². Beyond this threshold, no significant further increase in defect density is detected. For subsequent Ga intercalation experiments, only samples exhibiting a defect density below 4 × 10¹¹ cm⁻² were selected to ensure optimal intercalation conditions. Defect density was calculated as proposed by Cançado et al. [1].

[1] Cançado LG, Jorio A, Ferreira EH, Stavale F, Achete CA, Capaz RB, Moutinho MV, Lombardo A, Kulmala TS, Ferrari AC. Quantifying defects in graphene via Raman spectroscopy at different excitation energies. Nano Lett. 2011, 11(8):3190-6.

1.
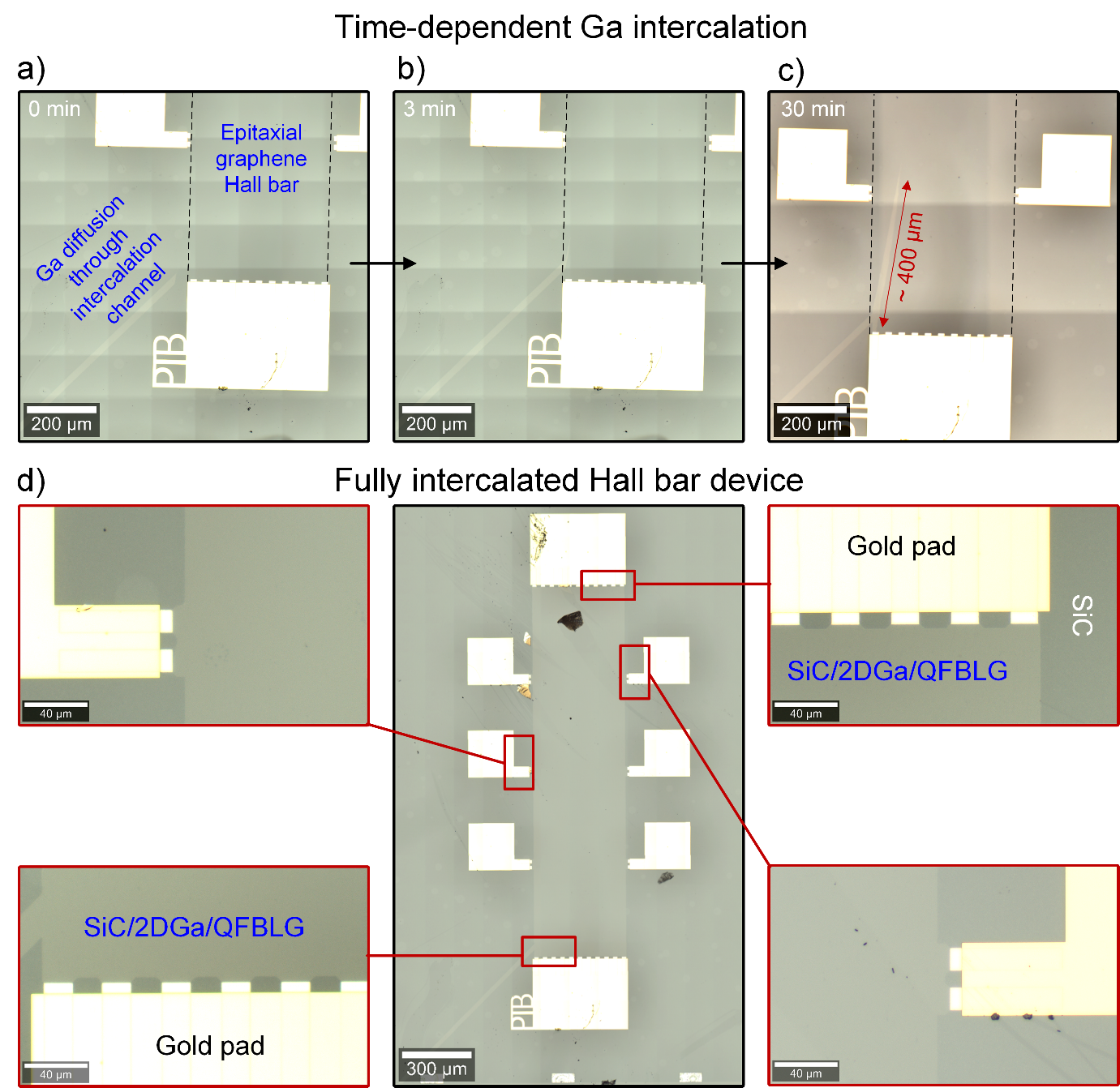
**Monitoring of Ga-diffusion beneath QFBLG**

**Fig S.5** **a–c)** Selected frames from video V.1 illustrating the progression of Ga intercalation through the intercalation channel into the epitaxial graphene Hall bar over a time span of 30 minutes. The Ga intercalation front propagates from the Hall bar edge inward, covering a lateral distance of approximately 400 µm within 27 minutes. This corresponds to an average intercalation rate of ~14.8 µm/min. **d)** Optical micrographs of the SiC/2DGa/QFBLG-Hall bar demonstrate that Ga intercalation proceeds up to the gold pads, confirming that the entire Hall bar structure has been successfully intercalated with Ga.

1.
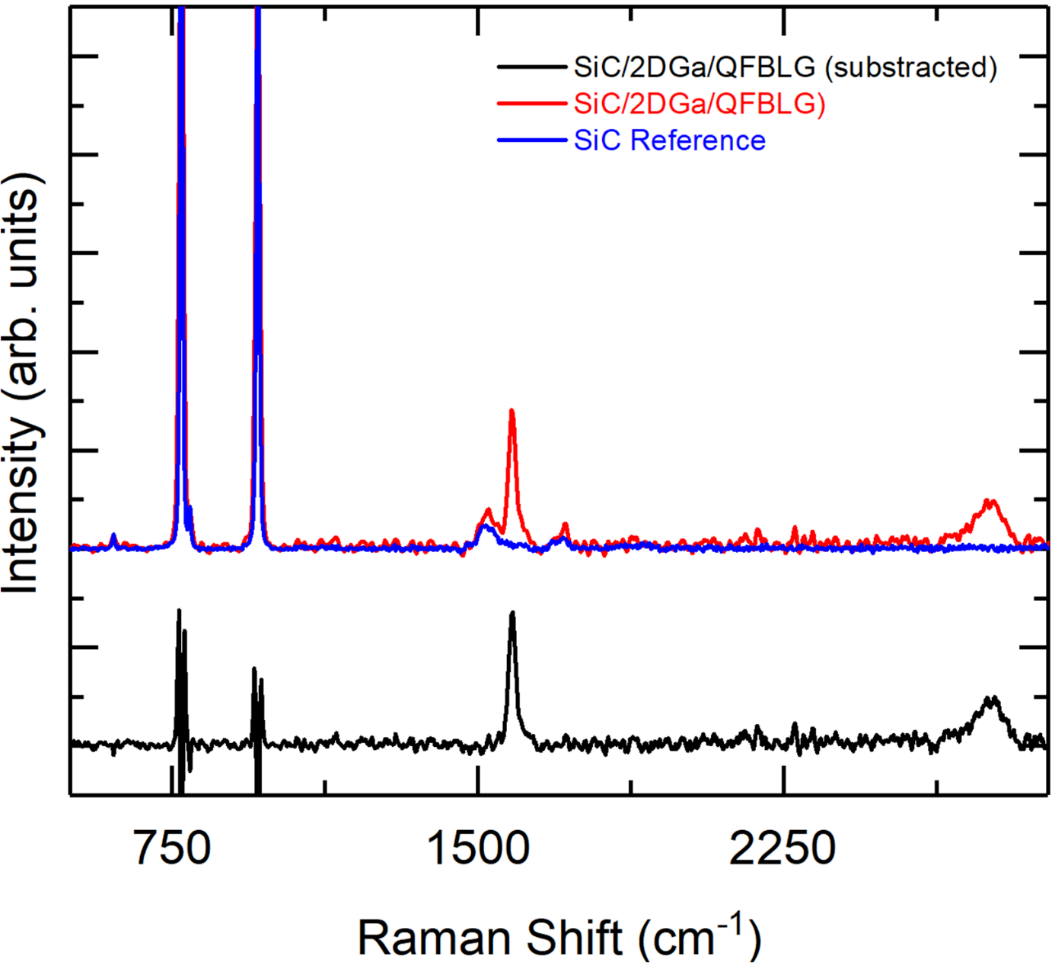
Raman spectrum of Ga-intercalated graphene and non-intercalated epitaxial graphene

**Fig S.6.** The Raman spectrum acquired from the Ga-intercalated graphene on the Hall bar area is shown in red. In this region, the graphene G peak is superimposed with the second-order Raman modes of the underlying SiC substrate. To isolate the G peak (approx. 1602 cm^-1^) from this spectral overlap, the spectrum is subtracted against a reference spectrum obtained from bare SiC (blue spectrum). The resulting black spectrum represents the corrected Raman signal of pure Ga-intercalated QFBLG. As expected, the subtraction procedure introduces spectral artifacts, most notably overshoot features around 750 cm⁻¹, which originate from residual SiC contributions. Despite these artifacts, the resulting spectrum clearly reveals the presence of the G peak of QFBLG while showing no detectable D peak. The absence of a D peak confirms the high structural quality of the QFBLG and indicates that no significant defect formation occurs during the Ga intercalation process.

1. **Raman mapping of 2DGa_(2)_ phase across intercalaton channel and Hall bar device**


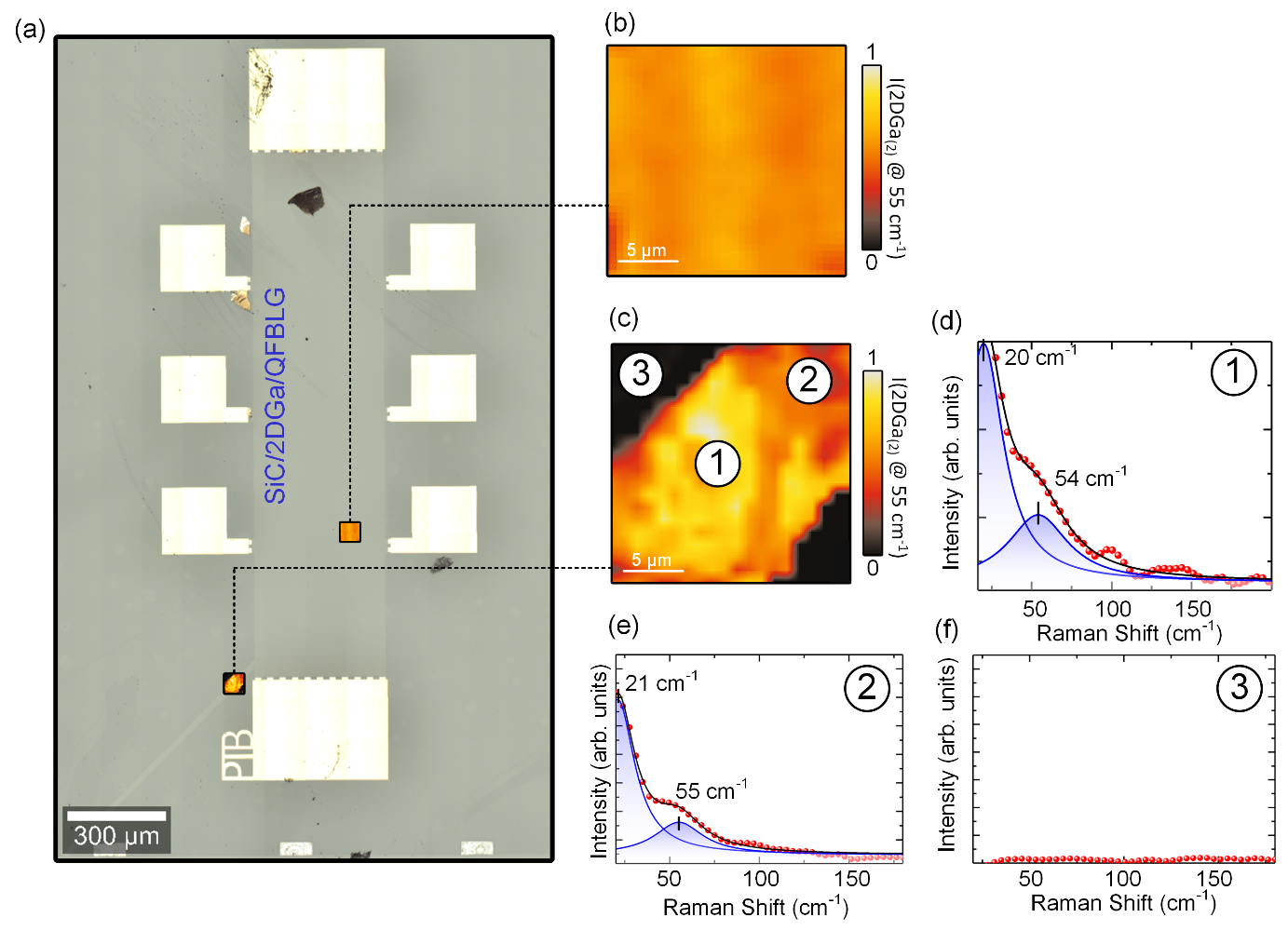


**Fig S.7.** Raman mapping was performed within the intercalation channel and across the Hall bar devices to identify the specific 2DGa phase. **a)** Optical micrograph of the SiC/2DGa/QFBLG Hall bar device indicating the locations where Raman mappings were acquired **b–c)** False-color images showing the spatial intensity distribution of the Ga phonon mode at 55 cm⁻¹, characteristic of the 2D-Ga_(2)_ phase. **d–e)** Raman spectra recorded at positions 1 and 2 within the intercalation channel. At both locations, the spectral features are dominated by the 2D-Ga_(2)_ phase. The 2D-Ga_(1)_ phase typically identified by a phonon mode at 109 cm⁻¹ was not detected in either the intercalation channel or the Hall bar area. **f)** Outside the intercalation channel, no Raman signal corresponding to the 2D-Ga_(2)_ phase is observed. Moreover, no enhanced intensity of the 2D-Ga_(2)_ phonon mode is detected at the channel edges, indicating that there is no accumulation of gallium at the boundaries of the intercalation front.

1. **Scanning tunneling microscopy for determination of Ga layer thickness across the epitaxial graphene-SiC/2DGa/QFBLG transition zone**


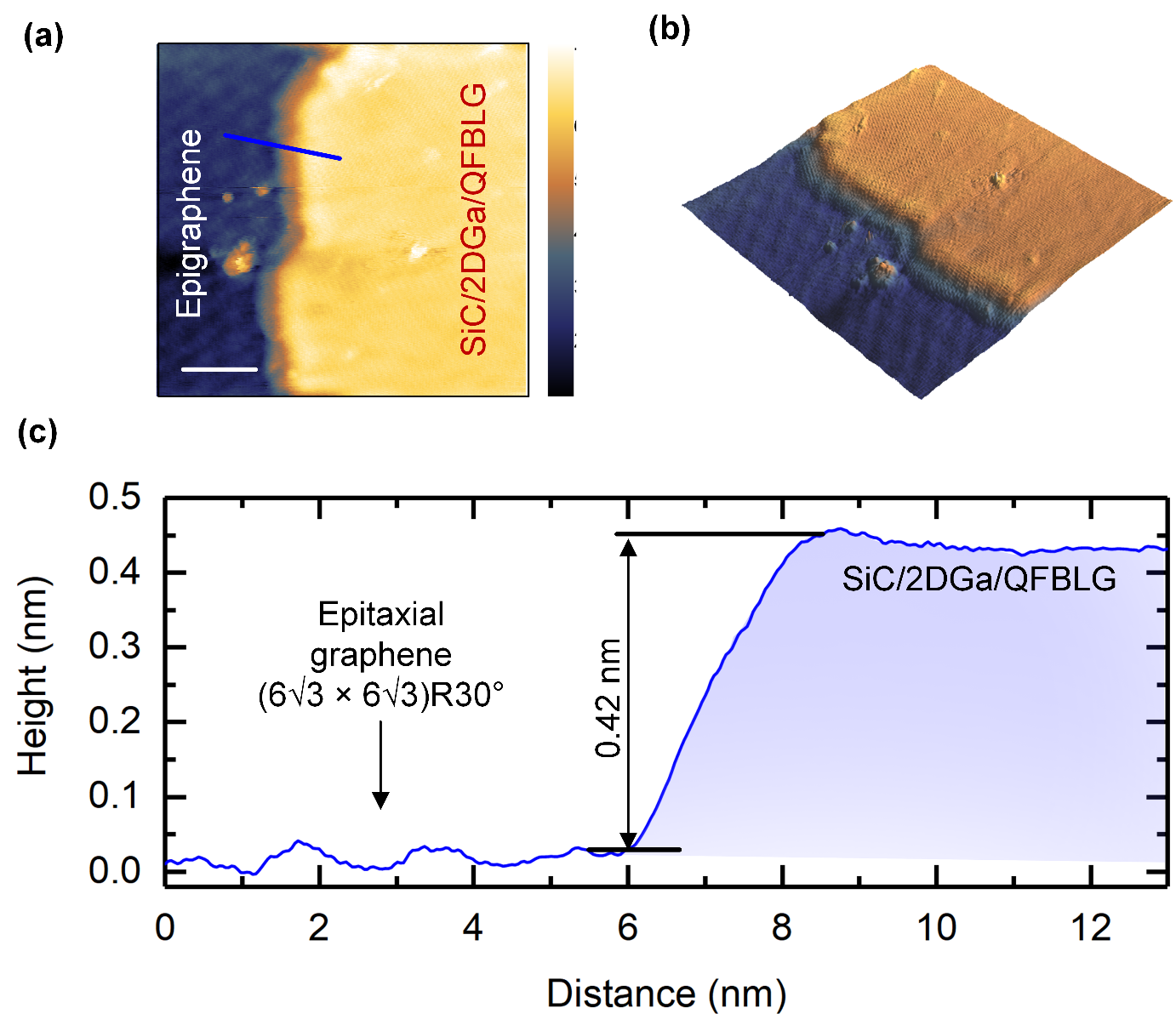


**Fig S.8** **a–b)** Scanning tunneling microscopy (STM) measurements at the interface between Ga-intercalated QFBLG (highlighted in yellow) and the adjacent epitaxial graphene region (highlighted in blue). **c)** Line profile acquired across the transition from epitaxial graphene to 2DGa/QFBLG, as indicated in (a), reveals a step height of approximately 0.42 nm, corresponding to the thickness of the intercalated Ga layer.

1. **Bade****r analysis of epitaxial graphene and SiC/2DGa/QFBLG**

**SiC/Ga/QFBLG**


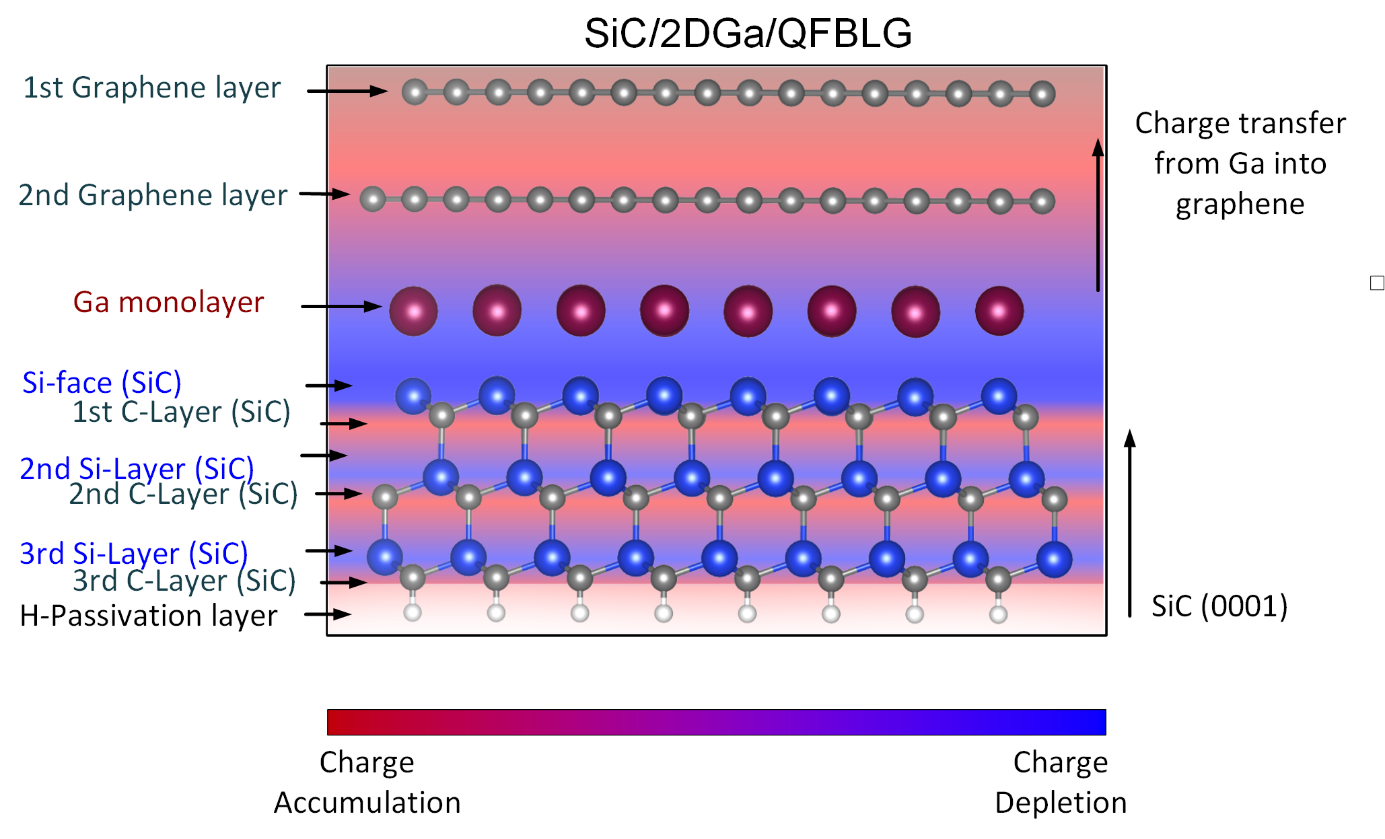


**Fig S.9.** Schematic illustration of the DFT supercell used for Bader charge analysis: QFBLG atop a Ga monolayer on SiC(0001) with bottom H passivation. Layers are labeled (first and second graphene, Ga monolayer, Si-face, deeper Si–C bilayers). The background color qualitatively depicts charge accumulation (red) and depletion (blue) and has no quantitative physical meaning.

The supercell was built by expanding the (√3 × √3)R30° SiC(0001) cell to suppress adatom–image interactions, with lateral size about (13.84 × 15.97) Å², cell height about 16 Å, and about 12 Å of vacuum. Lattice relaxation and SCF convergence were achieved using the Perdew–Burke–Ernzerhof parametrization of the generalized gradient approximation (GGA-PBE) for the exchange–correlation functional. Projector augmented wave (PAW) pseudopotentials and a van der Waals correction using Grimme D2 were employed. The lattice relaxation used a plane-wave energy cutoff of 816 eV and a force convergence threshold of 0.003 eV/Å.

Bader charge calculation was first performed by computing Bader volumes and partitioning the charge density within the supercell [2]. The atomic net charge *q*_net_ is defined as

*q*_net_ = *Z* – *N_e_*_,frozen core_ − *N*_Bader ,_

where *Z* is the atomic number, *N_e_*_,frozen core_ is the number of frozen core electrons for the atom in

[2] W. Tang, E. Sanville, and G. Henkelman [A grid-based Bader analysis algorithm without lattice bias](https://theory.cm.utexas.edu/henkelman/code/bader/download/tang09_084204.pdf), J. Phys. Condens. Matter **21**, 084204 (2009).

the employed PAW potential, and *N*_Bader_ is the valence-electron population integrated within the Bader volume. Here, a negative (positive) *q*_net_ indicates electron accumulation (depletion). The average atomic net charge within the layer has been calculated using:

${\bar{\boldsymbol{q}}}_{net} = \frac{{\sum q}_{net}}{i}$ ,

where *i* denotes the total number of atoms of the considered layer within the supercell.

The net charge of a given atomic layer *Q*_L_​ (e.g., graphene, Ga monolayer, Si face) was obtained by summing the atomic net charges of all atoms assigned to that layer:

*Q*_Layer_ = $\sum q_{net}$

Finally, the Bader net charge density *n*_Layer_ of the layer was obtained by:

$n_{Layer}= \frac{Q_{Layer}}{A} \times{10}^{16} ({cm}^{-2})$ ,

where *A* denotes the in-plane area of the supercell (*A* ≈ 221 Å²) derived from the lattice vectors. Charge neutrality was checked by verifying the total sum of atomic net charges Σ*q*_net_ ≈ 0. However, the charge neutrality within the Bader partitioning leaves a residual of 0.007 *e* per atom within the supercell. This likely reflects minor residual convergence limits in the lattice relaxation of the very large supercell used and the applied parameters. The residual is small but should be accounted for as an uncertainty contribution.

**Table S1. Bader charge results of SiC/Ga/QFBLG supercell**

| Layer | Averaged atomic net charge, $\bar{\boldsymbol{q}}$_net_ | Bader net charge density per layer,  *n*_Layer_ |
| --- | --- | --- |
|  | (*e* per atom) | cm^-2^ |
| 1st Graphene layer | -0.002 | -0.68 × 10^13^ |
| 2nd Graphene layer | -0.014 | -4.47 × 10^13^ |
| Ga Monolayer | +0.078 | +9.48 × 10^13^ |
| Si-Face (SiC) | +1.945 | +2.38 × 10^15^ |
| 1st C-Layer (SiC) | -2.615 | -3.19 × 10^15^ |
| 2nd Si-Layer (SiC) | +2.631 | +3.22 × 10^15^ |
| 2nd C-Layer (SiC) | -2.659 | -3.25 × 10^15^ |
| 3rd Si-Layer (SiC) | +2.657 | +3.25 × 10^15^ |
| 3rd C-Layer (SiC) | -2.014 | -2.46 × 10^15^ |
| H-Layer (Passivation) | +0.019 | +2.39 × 10^13^ |

1. **Critical current *I*c measured in superconducting SiC/2DGa/QBFLG Hall bar**


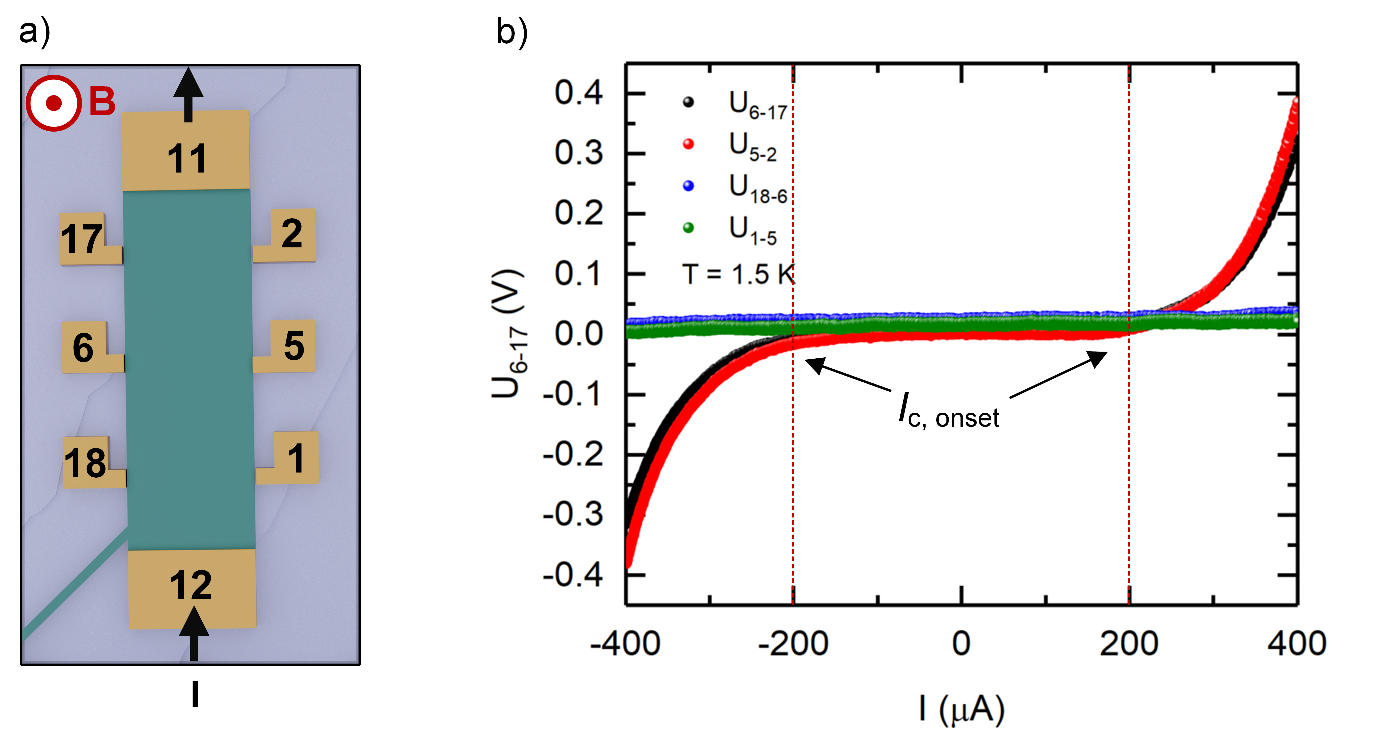


U (V)

**Fig S.10** **a)** Schematic representation of the SiC/2DGa/QFBLG Hall bar device. The bias current I is applied between contacts 12 and 11. Longitudinal voltage (U6-17, U5-2, U18-6, U1-5) has been probed by current sweeping at 1.5 K **b)** Current–voltage (I–U) characteristics. Longitudinal zero-voltage plateau between -200 and 200 μA indicates the superconducting phase of SiC/2DGa/QFBLG. The onset of finite voltage beyond I_c,onset_ ≈ ± 200 μA marks the breakdown of superconductivity.

1. **
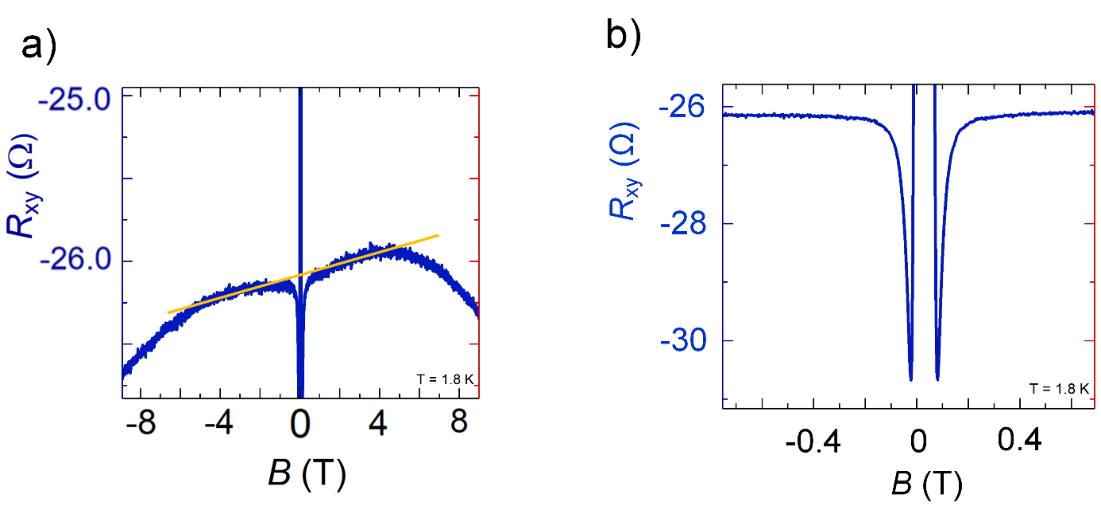
*R*_xy_(H) in SiC/2DGa/QFBLG: symmetric and antisymmetric in-field components**

**Fig S.9** **a)** Transverse resistance *R*_xy_(B) characteristics of the SiC/2DGa/QFBLG system measured under magnetic field sweeps between ±8 T. The Ga-intercalated sample exhibits both symmetric and antisymmetric components in the field-dependent *R*_xy_(B), with an approximately constant negative transverse resistance of ~26 Ω across the entire field range. This seemingly anomalous behavior is likely attributed to inhomogeneous current distribution and current jetting effects, which result in admixture of longitudinal and transverse resistance components. **b)** A distinct peak anomaly is observed at *B* = *B*_c2_ ≈ 100 mT, indicative of a transition related to the upper critical field of the superconducting phase.
